# 3′ UTR-mediated regulation of a protein chaperone by the *pspA* mRNA in *Streptococcus pneumoniae*

**DOI:** 10.1093/nar/gkag481

**Published:** 2026-05-14

**Authors:** Jens S Pettersen, Anette Lund, Finn Kirpekar, Nikolaj V Hansen, Sine L Svenningsen, James C Paton, Jakob Møller-Jensen, Mikkel G Jørgensen

**Affiliations:** Department of Biochemistry and Molecular Biology, University of Southern Denmark, 5230Odense,Denmark; Department of Biochemistry and Molecular Biology, University of Southern Denmark, 5230Odense,Denmark; Department of Biochemistry and Molecular Biology, University of Southern Denmark, 5230Odense,Denmark; Department of Biochemistry and Molecular Biology, University of Southern Denmark, 5230Odense,Denmark; Department of Biology, University of Copenhagen, 2200Copenhagen,Denmark; Research Centre for Infectious Diseases, Department of Molecular and Biomedical Science, University of Adelaide, Adelaide, SA 5005,Australia; Department of Biochemistry and Molecular Biology, University of Southern Denmark, 5230Odense,Denmark; Department of Biochemistry and Molecular Biology, University of Southern Denmark, 5230Odense,Denmark

## Abstract

RNA-mediated control of virulence gene expression plays a crucial role in many pathogenic bacteria. However, our understanding of these processes in *Streptococcus pneumoniae*, a major human pathogen, remains limited. Here we discover a novel regulatory element located in the 3′-untranslated region (3′ UTR) of the mRNA encoding a major pneumococcal virulence factor, pneumococcal surface protein A (PspA). Quantitative proteomics and western blot analysis reveal that this 3′ UTR acts as a *trans*-acting riboregulatory element, modulating the expression of the protein chaperone, Caseinolytic protease L (ClpL), in a temperature-dependent manner. We show that it is the full-length *pspA* mRNA, and not the *pspA*-3′-located processed F5 RNA, which is involved in the regulation of ClpL expression, with the sRNA-interacting exoribonuclease Cbf1 playing an important role. Furthermore, complement deposition assays show that the regulatory *pspA*-3′ UTR contributes to inhibition of complement C3 deposition in a PspA-independent and temperature-dependent manner. This discovery adds a new dimension to our understanding of PspA’s role in bacterial virulence, highlighting an intricate layer of RNA-mediated regulation that contributes to the pathogenicity of *S. pneumoniae*.

## Graphical Abstract

**Graphical Abstract:**
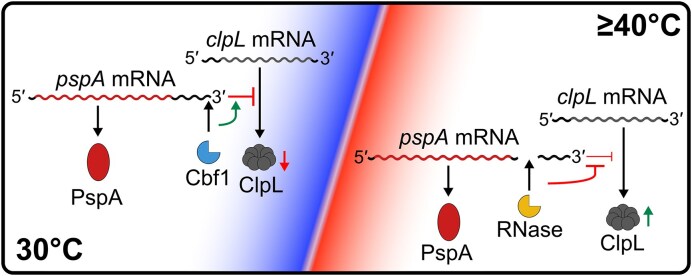

## Introduction

Small non-coding RNAs (sRNAs) are crucial riboregulatory elements in bacteria, modulating gene expression by base-pairing with target RNAs through various mechanisms [1, 2]. In pathogenic bacteria, numerous sRNAs essential for fine-tuning virulence gene expression have been identified over the past decades [3–5]. These discoveries have expanded our understanding of the complex regulatory networks that allow pathogens to adapt to the human host. Despite *Streptococcus pneumoniae* (pneumococcus) being a leading cause of severe infectious diseases such as pneumonia, sepsis, and meningitis [6], RNA-mediated gene regulation in this Gram-positive pathogen remains poorly understood. Pneumococcus can infect multiple human niches, including the nasopharynx, middle ear, lung, bloodstream, and the meninges [7], indicating that its adaptation requires tight regulation of virulence genes [8–11], possibly mediated by RNA-based mechanisms.

While several studies have examined the pneumococcal sRNA transcriptome, identifying more than 100 sRNAs in different strains [12–14], only a few have been thoroughly characterized. Among them are the cia-dependent small RNAs (csRNAs), a group of five homologous sRNAs involved with competence control [15]. More recently, a 3′-untranslated region (UTR)-derived sRNA, PlyT, was identified as a regulator of pneumolysin (Ply) synthesis, inhibiting Ply production in anaerobic conditions and contributing to brain invasion [16].

A genome-wide analysis by Mann *et al*. identified 89 sRNAs in *S. pneumoniae* TIGR4, with 18, 26, and 28 sRNAs predicted to contribute to pathogenesis in the blood, nasopharynx, and lungs of mice, respectively [13]. Among these, F5 was identified as an important sRNA for pneumococcal survival in the bloodstream of mice. In this study, we further investigate F5 to explore its functional role. F5 is located downstream of the gene encoding pneumococcal surface protein A (PspA), a major virulence factor on the pneumococcal surface that plays a significant role in immune evasion by inhibiting complement deposition and neutralizing lactoferrin (LF) [17–21]. Beyond immune evasion, PspA also binds to host glyceraldehyde-3-phosphate dehydrogenase (GAPDH) to mediate adherence to dying lung epithelial cells and enhances virulence by binding lactate dehydrogenase (LDH), promoting local lactate production [22, 23].

This work reveals that F5 is a 3′-UTR-located RNA element of the *pspA* mRNA, adding a novel riboregulatory function to the repertoire of the multifunctional PspA [24]. We demonstrate that the 3′ UTR functions as a riboregulatory element within the context of the primary transcript, thus functioning as a long *trans*-acting regulatory mRNA. Specifically, the *pspA* 3′ UTR post-transcriptionally regulates the expression of Caseinolytic protease L (ClpL) in a temperature-dependent manner, a process facilitated by the sRNA-stabilizing 3′-5′ exonuclease, Cbf1.

## Materials and methods

### Bacterial growth conditions and transformation

The serotype 2 strain *Streptococcus pneumoniae* D39 and derivatives were grown in the semi-defined C + Y medium or on Columbia agar plates supplemented with 2% (v/v) defibrinated horse-blood (SSI-Denmark, 23 699) at 37°C with 5% CO_2_, unless stated otherwise. Transformation was performed by inducing pre-competent pneumococcal cells (stocks of 10-fold concentrated cultures of OD600 of 0.1 in C + Y medium (pH 7)) with 0.2 µg/ml competence-stimulating peptide (CSP) for 12 min at 37°C. Following this, DNA was added for uptake and homologous recombination for 20 min at 30°C. Finally, transformations were incubated at 37°C to allow expression of antibiotic resistance genes for at least 1.5 h before spreading on Columbia blood agar plates and incubating overnight. For selection, chloramphenicol (cml), kanamycin (kan), spectinomycin (spc), or streptomycin (str) was added in the following concentrations: 4.5 µg/ml, 250 µg/ml, 100 µg/ml, and 200 µg/ml, respectively. Linear PCR products with homologous overhangs of approximately 1000 base pairs were used for transformation. For ectopic overexpression, a chromosomal expression platform (CEP) with a constitutive active promoter (P3) was used, as described by Sorg *et al*. [25]. A full list of strains constructed for this study is in the supplementary material (Supplementary Table S1). Oligos used for amplification of PCR products are listed in Supplementary Table S2. Following confirmation by PCR, strains were sent for Sanger sequencing at Eurofins. Growth experiments were carried out in 96-well plates (SARSTEDT, 83.3924.500) using a plate reader (Synergy^TM^ H1 multimode reader, BioTek).

### RNA extraction

RNA was extracted from pneumococcal cell pellets by phenol-chloroform extraction. First, pellets were resuspended in cold solution 1 (10 mM Na-Citrate, 10 mM Na-acetate, pH 4.5, and 2 mM EDTA) and added to a mixture of 150 μL solution 2 (10 mM Na-acetate, pH 4.5, and 2% sodium dodecyl sulfate (SDS)), 700 μL acidic phenol (pH 4.5), and 300 μL chloroform. Tubes were then inverted and heated at 80°C for 4 min, followed by cooling on ice. Subsequently, tubes were centrifuged at 10 000 × g for 5 min, and the aqueous phase was transferred to 96% ethanol with Na-acetate (37.5 mM) for precipitation. RNA was then pelleted by centrifugation (20 000 × g for 45 min) and washed in ice-cold ethanol. RNA pellets were resuspended in RNase-free H_2_O.

### Northern blot analysis

Unless otherwise stated, 10 µg of RNA was mixed with formamide loading dye and separated on a denaturing polyacrylamide gel (4.5 or 8%) for 2 h at 300 V. Separated RNA was blotted onto a Zeta-probe nylon membrane (Bio-Rad, 1 620 159) using a semi-dry transfer unit for 1 h at 400 mA. RNA was cross-linked to the membrane by UV radiation. Oligonucleotide probes (Supplementary Table S2) were 5′-labeled with ^32^P-ATP using T4-polynucleotide kinase (New England Biolabs, M0201). Membranes were pre-hybridized in hybridization buffer (PerfectHyb™ Plus, Sigma-Aldrich, H7033) for 10 min before adding 5′-labeled oligos and incubating overnight, all at 42°C. Probed membranes were washed for 10 min each in 2 × Saline-Sodium Citrate (SCC) containing 0.1% SDS and then in 0.5 × SCC with 0.1% SDS, wrapped in plastic film, and placed on a storage phosphor screen. Bands were visualized on a Typhoon FLA9500 (GE Healthcare). Images were analyzed using ImageJ software. Brightness adjustments were applied uniformly to the entire image to enhance visibility.

### Rapid amplification of cDNA ends (5′ RACE) analysis

RNA was isolated from mid-exponential and heat-stressed (42°C for 30 min) pneumococcus. RNA samples were treated with DNase I (New England BioLabs, M0303) (4 units) for 20 min at 37 °C and purified again by phenol-chloroform extraction. 12 μg of RNA were dissolved in H_2_O and mixed with 1x Tobacco Acid Pyrophosphatase (TAP) buffer (500mM sodium acetate (pH 6.0), 10mM EDTA, 1% β-mercaptoethanol, 0.1% Triton X-100) and RNase inhibitor (20 U, New England BioLabs, M0314). One part of the sample was treated with the Cap-Clip™ Acid Pyrophosphatase (10 U, Cellscript, C-CC15011H) for 30 min at 37°C, and the other was left untreated. 500 pmol of an RNA adapter oligonucleotide was added to each tube, and phenol-chloroform extraction was carried out to remove enzymes. RNA Adapter was ligated with T4 RNA ligase (20 U, New England BioLabs, M0204) overnight at 17°C. Reverse transcription was performed with the Maxima Reverse Transcriptase (200 U, ThermoFisher Scientific, EP0741) and the F5-specific primer reverse primer (‘F5,’ Supplementary Table S2). Amplification of cDNA was carried out with PCR using the F5-specific reverse primer, and an RNA adapter-specific forward primer (‘5RACE_adapter_F’, Supplementary Table S2). The amplified DNA was separated on a 2% agarose gel, and the bands of interest were cut out, purified, and sent for sequencing at Eurofins.

### Rifampicin RNA stability assay

Cultures of *S. pneumoniae* D39 WT and Δ*3UTR* were grown in C + Y medium at 30°C, 37°C, or 40°C to an OD_600_ of 0.2 (n = 3) before transcription was blocked by 500 µg/ml rifampicin. At 0, 2, 4, 8, and 16 min after rifampicin addition, 8 ml of culture was snap frozen in liquid nitrogen. RNA was isolated as described above, and reverse-transcription quantitative PCR (RT-qPCR) was used to quantify transcript levels. Specifically, 1 µg RNA from each condition was treated with DNase I (New England BioLabs, M0303) prior to cDNA synthesis using the High-Capacity cDNA Reverse Transcription Kit (Applied Biosystems™, Thermo Fisher, 4 368 814). Real-time quantitative PCR was carried out with RealQ Plus 2x Master Mix Green (Ampliqon, A323402) and gene-specific primers on a LightCycler 480 System. An exponential decay function was fitted to the data sets by non-linear regression using the *nls* function from the R stats package.

### In vitro transcription and electrophoretic mobility shift assays

In vitro transcripts used for the gel shift assays were synthesized by T7 transcription using the MEGAscript™ T7 Kit. Templates for T7 transcription were constructed by PCR using a forward primer with the T7 promoter in the overhang. T7 transcripts were separated by PAGE, and the band of interest was eluted from the gel by electro-elution using GeBAflex tubes (Scienova). RNA was precipitated in ethanol with Na-acetate (37.5 mM). RNA transcripts were 5′-labeled with [γ-32P] ATP using T4-polynucleotide kinase (NEB). Binding reactions for gel shift assays were carried out in 1x Binding buffer (10 mM Tris pH 8, 75 mM KCl, 1 mM MgCl_2_, 0.5 mM DTT) with yeast tRNA as nonspecific competitor using 2 nM 32P-labeled RNA, 0–2000 nM unlabeled target RNA, and/or 0–2 µM Cbf1^3xFLAG^ protein, dependent on the experiment. Different incubation times for binding reactions were tested. Unless otherwise stated, they were as follows: for RNA-RNA gel shift assays, binding reactions were incubated at 80°C for 2 min, placed on ice, and then incubated at 37°C for 30 min. For RNA-Cbf1 gel shift assays, 32P-labeled RNA was incubated at 80°C for 2 min, placed on ice, and incubated at 37°C for 10 min before adding Cbf1 and incubating for another 20 min. For RNA-RNA-Cbf1 gel shifts, 32P-labeled RNA was incubated at 80°C for 2 min, placed on ice, incubated at 37°C for 15 min with Cbf1^3xFLAG^, followed by 15 min incubation with target RNA. Binding reactions were separated by native gel electrophoresis on 5% polyacrylamide gels at 4°C in 0.5 × TBE, dried down, and placed in a storage phosphor screen. Gels were imaged using a Typhoon scanner (GE Healthcare).

### Western blot analysis

Samples for western blot analysis were prepared by harvesting 1 ml culture and pelleted by centrifugation (20 000 × g for 5 min). Pellets were resuspended in 1xSDS sample buffer (80 mM Tris, pH 6.8, 2% SDS, 10% glycerol, 0.02% bromophenol blue, and 0.1 M DTT) to a concentration of 5 × 10^5^ cells/µl (assuming OD_600_ 0.1 = 10^7^ cells/ml) and heated at 95°C for 5 min. Samples (10 µl) were separated on a NuPAGE 4–12%, bis-tris gel (Invitrogen™, Thermo Fisher, NP0321 or NP0322) for 45 min at 200 V in 1xMOPS running buffer. Separated proteins were transferred to a polyvinylidene difluoride (PVDF) membrane in a wet tank transfer unit for 1 h at 300 mA. Membrane was stained with an amido black solution (0.1% Amido Black 10B, 40% methanol, 10% acetic acid) for 5 min, followed by de-staining (1–3 min in 20% methanol, 7.5% acetic acid). Blocking of the membrane was carried out using 1 x PBS-Tween (0.1% Tween-20) with 5% w/v nonfat dry milk. Membrane was incubated with primary antibodies (diluted 1:10 000 in 1xPBS-Tween with 2% dry milk) for 1 h or overnight, and secondary antibodies (diluted 1:4000) for 1 h. Primary antibodies used were as follows: the monoclonal mouse anti-FLAG M2 (Sigma Aldrich, F3165), polyclonal goat anti-PspA (gift from PhD Edmund Loh, Karolinska Institute), and polyclonal rabbit anti-human C3c (Agilent / Dako, A0062). Secondary antibodies used were the horseradish peroxidase (HRP)-conjugated polyclonal goat/rabbit anti-rabbit/mouse/goat secondary antibodies (Agilent / Dako, P0447, P0448, P0449). Immobilon Forte (Milipore, Sigma Aldrich, WBLUF) was used as an HRP substrate, and detection of chemiluminescent signals was carried out on an Amersham ImageQuant 800. Images were analyzed using ImageJ software. Brightness adjustments were applied uniformly to the entire image to enhance visibility.

### Subcellular fractionation of *Streptococcus pneumoniae*

A protocol similar to that described in [26] was used for pneumococcus fractionation. Briefly, pellets were incubated in a cell wall digestion buffer (10 mM Tris pH 7.5, 30% sucrose, 300 U/ml mutanolysin, and 1 mg/ml lysozyme) at 37°C for 2 h with rocking. Protoplast and cell wall fraction were separated by centrifugation at 14 000 × g for 15 min, at 4°C. Protoplast pellet was resuspended in 1xSDS sample buffer, and proteins from the cell wall supernatant were precipitated with 10% (v/v) trichloroacetic acid (TCA) at 4°C for 1 h. Proteins were pelleted by centrifugation (14 000 × g for 10 min) and washed in ice-cold acetone. Protein pellets were resuspended in 1xSDS sample buffer and stored at −20°C.

### Protein purification and digestion

*S. pneumoniae* D39 wild type, Δ*3UTR*, and Δ*3UTR* CEP:*3UTR* cultures were grown in triplicate in C + Y medium at 40°C to OD_600_ 0.5. Cultures were harvested by centrifugation (10 000 × g for 10 min, at 4°C), and pellets were resuspended in a Tris buffer (20 mM Tris-HCl, pH 7.5) with protease inhibitor cocktail (Sigma Aldrich, P8465). Resuspended cells were lysed by two passes through a French pressure cell. Lysates were cleared by centrifugation (16 000 × g for 30 min, at 4°C), and total protein concentration in each sample was measured using the Bio-Rad Protein Assay (Bio-Rad, 5 000 002). 200 µg protein extract was adjusted to 30 mM triethylammonium bicarbonate (TEAB) (pH 8.5) reduced with 5 mM DTT for 30 min at 37°C in the dark, followed by the alkylation with iodoacetamide (45 mM final concentration) under identical incubation conditions.

The proteins were predigested with 0.005 AU lysyl endopeptidase (FUJIFILM Wako Pure Chemical Corporation) at 37°C for 3 h, and the concentration of urea was subsequently diluted to < 1.5 M by adding 20 mM TEAB (pH 8.0). Protein was digested with in-house methylated trypsin [27] in an enzyme:protein ratio of 1:50 at 37°C overnight. To stop the digestion, samples were acidified by adjusting to 1% trifluoroacetic acid (TFA) followed by centrifugation at 20 000 × g for 15 min. The peptides were desalted by self-packed columns with a small plug of 3M™ Empore™ C8 (3M Bioanalytical) at the end of a 200 µl tip and packed with POROS™ 50 R2 Reversed-Phase resin (Thermo Fisher Scientific), followed by desalting with 3M™ Empore™ C18 material (3M Bioanalytical) packed with OLIGO™ R3 Reversed-Phase resin (ThermoFisher Scientific). Columns were washed with 0.1% TFA, and the peptides were eluted with 60% acetonitrile/0.1% TFA, dried by vacuum centrifugation, and resuspended in 50 µl 50 mM TEAB buffer.

### TMT 10-plex labelling

Around 50 µg peptides from each sample were labeled with 0.25mg of a tag from a TMT Label reagent set (TMT10plex™ Isobaric Label Reagent Set from ThermoFisher Scientific, #90 110). According to the manufacturer’s protocol. The experiment was carried out in three biological replicates with one technical replicate in each, as follows. To quench the labeling reaction, 5.7 µl of 5% hydroxylamine was added to each sample and incubated for 15 min at RT. The samples were mixed in a 1:1:1:1:1:1:1:1:1 ratio based on the median values of the identified labeled peptides from a combined aliquot pre-run on LC-MS/MS and searched in Proteome Discoverer 2.4 (Thermo Fisher Scientific). The samples were acidified to 1% TFA, dried by vacuum centrifugation, and resuspended in 200 µl 0.1% TFA for desalting by self-packed columns as described above. The sample was then resuspended in 40 µl 20 mM NH_4_COOH, pH 9.3.

### High pH UPLC fractionation

The TMT labeled peptides were fractionated on a Dionex Ultimate 3000 HPLC^+^ Focused system (Thermo Fisher Scientific, Odense, Denmark) with a *nanoEase M/Z Peptide C18* column (Waters). The mobile phase consisted of solvent A (20 mM NH_4_COOH, pH 9.3) and solvent B (80% ACN, 20% solvent A). The flow rate used for separation was 5 µl/min, and the gradient was as follows: 2–40% solvent B in 85 min, 40–50% solvent B in 32 min, 50–95% solvent B in 5 min, and 95% solvent B for 10 min. The HPLC fractions were separated into 20 fractions and dried by vacuum centrifugation.

### LC-MS/MS analysis

The fractionated peptides were resuspended in 10 µl solvent A (0.1% formic acid (FA)) and automatically loaded on a two-column EASY-nlC system (Thermo Fisher Scientific). The pre-column was a 3 cm long fused silica trap column (100 µm inner diameter) with a fritted end and packed in-house with Reprosil-Pur C18-AQ 5 µM particles (Dr. Maisch GmbH, Germany), and the analytical column was a 20 cm long fused silica trap column (75 µm inner diameter) and packed in-house with Reprosil-Pur C18-AQ 3 µM particles (Dr. Maisch GmbH, Germany). The peptides were eluted at a constant flowrate of 250 nl/min with gradient going from 95% solvent A to 100% solvent B (95% ACN, 0.1% FA) as follows: 5–10% solvent B in 8 min, 10–34% solvent B in 120 min, 34–50% solvent B in 10 min, 50–100% solvent B in 5 min and 100% solvent B for 8 min. The EASY-nLC was connected online to a Q Exactive HF mass spectrometer (Thermo Fisher Scientific). The MS analysis was performed in data-dependent acquisition mode at a high resolution of 120 000 (m/z 200) with the mass range of 350–1600 m/z. The target value was set to 3.00E + 06 with a maximum injection time of 100 ms. The 15 most abundant ions were selected from the MS with an isolation width of 1.2 m/z for fragmentation in the HCD collision cell using a collision energy of 34%. The fragmentation was performed at a high resolution of 30 000 (m/z 200) for a target value of 1.00E + 05 with a maximum injection time of 120 ms. 10% ammonia solution was placed underneath the needle in order to reduce the charge state of the labeled peptides [52].

### Protein identification and quantification

The raw MS data files were processed using the Proteome Discoverer software version 2.4 (Thermo Fisher Scientific) and searched against a *Streptococcus pneumoniae* protein database https://www.ncbi.nlm.nih.gov/genomes/GenomesGroup.cgi?taxid=373153 (downloaded August 2020) using the Mascot search engine (Matrix Science). Trypsin was selected as the enzyme, and a maximum of three missed cleavages were allowed. The precursor mass tolerance was set to 10 ppm and the fragment mass tolerance was set to 0.05 Da. TMT 10-plex on peptide *N*-terminals and lysine side chains as well as oxidation of methionines were selected as dynamic modification and carbamidomethyl of cysteines was selected as a static modification. To correct experimental bias, the normalization to the protein median of each sample was used. Only proteins identified with at least two unique peptides were analyzed further. Differential expression analysis was carried out using the Bioconductor package ‘DEP’ in R. An adjusted p-value of < 0.05, and a fold change of 1.5 (log2 FC > 0.58) was used as cut-off values for differentially expressed proteins.

### RNA-sequencing and data analysis

RNA was purified from the same cultures described in the section for quantitative proteomics. Ribosomal RNA was depleted from total RNA using NEBNext rRNA Depletion Kit (Bacteria) (New England BioLabs, E7850). Subsequent RNA-seq library preparation was carried out with NEBNext Ultra II Directional RNA Library Prep Kit for Illumina (New England Biolabs, E7760). Paired-end sequencing was performed on a NovaSeq 6000 System (Illumina). Raw paired-end reads were mapped to the *S. pneumoniae* D39 chromosome (GenBank: CP000410.2) with Bowtie2 using local alignment. Reads were counted inside features with featureCounts from the Subread package. Reads were assigned to features with largest overlap, and multimapping reads were counted using a fractional count (1/n, where n is the total number of alignments reported). Differential expression analysis was performed in R using EdgeR. Genes with a logFC > 1 and FDR < 0.05 were defined as significantly differentially expressed.

### Cbf1^3xFLAG^ purification and pull-down

To purify Cbf1 from *S. pneumoniae* D39, a 3xFLAG-tag mutant overexpressing Cbf1^3xFLAG^ from the CEP site (D39 CEP:*cbf1-3xflag*) was constructed. D39 CEP:*cbf1-3xflag* was grown in Brain Heart Infusion (BHI) broth medium (Merck, 53 286) at 37°C, 5% CO_2_ to OD_600_ 0.5 and harvested by centrifugation at 15 250 × g for 20 min, at 4°C. Pellets were resuspended in an immunoprecipitation (IP) buffer (50 mM Tris–HCl, pH 7.5, 150 mM KCl, 1 mM MgCl_2_, 1 mM CaCl_2_, 1 mM DTT) and lysed by French press. Lysate was cleared by centrifugation (16 000 × g for 30 min, at 4°C) and 0.01% (v/v) Triton X-100 was added. Cleared lysate was incubated with ANTI-FLAG® M2 Affinity Gel (Sigma Aldrich, A2220) overnight at 4°C. Lysate and beads were transferred to Poly-Prep® Chromatography Columns (Bio-Rad, 7 311 550), washed thoroughly with FLAG buffer I (400 mM NaCl, 1 mM EDTA, 0,1% Triton X-100 in 1xPBS) and II (400 mM NaCl, 1 mM EDTA, 0,01% Triton X-100 in 1 x PBS), and eluted with elution buffer (500 mM arginine, 500 mM NaCl). Ultra-centrifugation with Amicon Ultra Centrifugal Filter Units (Millipore, UFC801024) was used to exchange the elution buffer with a protein storage buffer (20 mM Tris–HCl, pH 7.5, 200 mM NaCl, 1 mM DTT, 10% glycerol). Protein samples were analyzed by SDS–PAGE and quantified with the Bio-Rad protein assay (Bio-Rad, 5 000 002) using a bovine serum albumin (BSA) standard. Protein samples were stored in aliquots at -80°C. In parallel with Cbf1^3xFLAG^ purification, a pull-down experiment with an identical immunoprecipitation setup was also carried out with duplicates cultures of D39 CEP:*cbf1-3xflag* grown OD_600_ 0.5 in BHI. RNA from the lysate (input), and eluate (output) fractions was purified by phenol–chloroform extraction, and transcript enrichment was quantified by RT-qPCR using the same protocol as described in the “Rifampicin RNA stability assay” section, using gene–specific primers listed in Supplementary Table S2.

### MS2-affinity purification

MS2 affinity purification was performed using a strain overexpressing *pspA* mRNA bearing an MS2 RNA aptamer fused to its 5′ end (Δ*pspA* CEP:*5′–MS2–pspA*), with an isogenic strain expressing untagged *pspA* mRNA (Δ*pspA* CEP:*pspA*) used as a control (Supplementary Table S1). Cultures were grown in C + Y medium at 30°C to mid–exponential phase (OD₆₀₀  0.6), and cells corresponding to 30 OD₆₀₀ units were harvested and resuspended in Buffer A (20 mM Tris–HCl pH 8.0, 150 mM KCl, 1 mM MgCl₂, 1 mM DTT). Cells were lysed by bead beating using Lysing Matrix B tubes (MP Biomedicals, 116 911 100) on a FastPrep–24 instrument (MP Biomedicals), and lysates were clarified by centrifugation at 15 000 × g for 15 min at 4°C. Polyprep columns were packed with 150 µL amylose resin, equilibrated with Buffer A, and incubated with 300 pmol MS2–MBP prior to loading of cleared lysates. Columns were washed three times with Buffer A and bound complexes were eluted with 0.9 mL Buffer A supplemented with 12 mM maltose. RNA was extracted from eluates by phenol–chloroform extraction, and transcript enrichment was quantified by RT–qPCR, using gene–specific primers listed in Supplementary Table S2.

### Complement deposition assay

*S. pneumoniae* cultures of D39 WT, Δ*3UTR*, Δ*pspA* and *pspA*-minimal strains were cultivated from in C + Y medium from a starting OD_600_ of 0.005 at either 30°C or 40°C (*n* = 3). Once the cultures reached an OD_600_ 0.05, 20% human plasma was added, followed by incubation for an additional 2 h. Samples were harvested by centrifugation (20 000 × g for 5 min) and cell pellets were washed three times in 1xPBS. Pellets were resuspended in 1 x SDS sample buffer (80 mM Tris, pH 6.8, 2% SDS, 10% glycerol, 0.02%, bromophenol blue and 0.1 M DTT) to a concentration of 5 × 10^5^ cells/µl and heated at 95°C for 5 min. Western blot was carried out as previously described with primary polyclonal rabbit anti-human C3c antibodies (Agilent/Dako) and secondary HRP-conjugated polyclonal goat anti-rabbit antibodies (Agilent/Dako). The PageRuler Plus Prestained Protein Ladder, 10–250 kDa (ThermoFisher Scientific, 26 620) was used for estimation of protein size. Statistical comparisons between groups of triplicates were performed using one-way analysis of variance (ANOVA), followed by Tukey’s post hoc test to identify specific group differences.

## Results

### F5 is a processed RNA fragment from the *pspA*-3′ UTR

**Figure 1.**
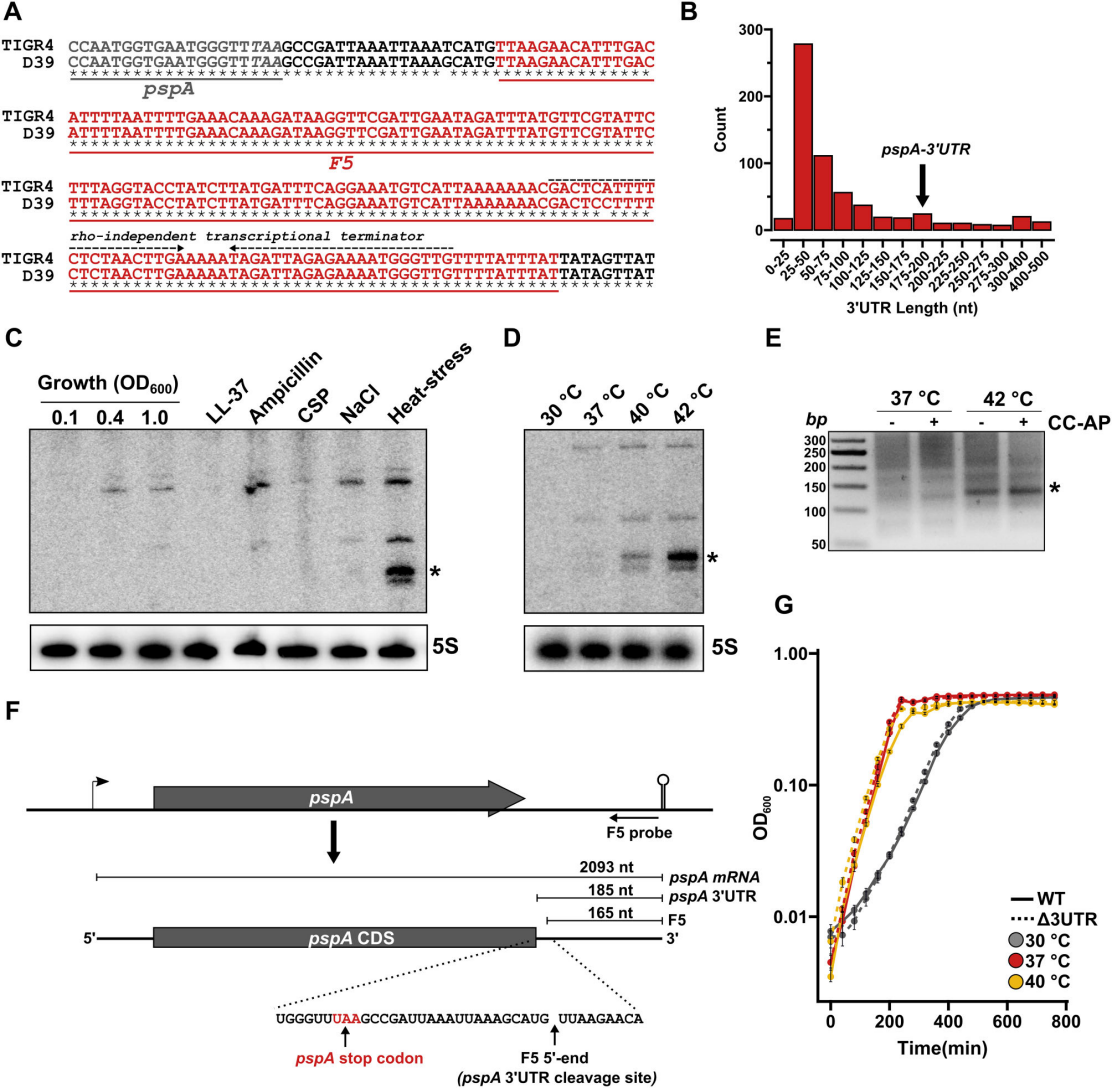
F5 is a *pspA* mRNA-derived RNA fragment. (**A**) The genetic region of F5 is conserved between the *S. pneumoniae* D39 and TIGR4 strains and is located directly downstream the *pspA* coding sequence (CDS) inside the 3′ UTR. (**B**) The distribution of pneumococcal 3′ UTR lengths reveal that the *pspA* 3′ UTR is longer than the majority of predicted 3′ UTRs in pneumococcus. UTR lengths were calculated based on transcript borders and CDS coordinates annotated in the D39V genome (GenBank: CP027540.1). (**C**) Using a F5 (*pspA*-3′ UTR)-specific probe, the F5 RNA levels were analyzed by northern blot analysis using RNA from standard growth conditions (OD_600_ 0.1, 0.4, and 1.0 in C + Y media at 37°C), and different stress- and infection-relevant conditions (exponentially growing pneumococci were exposed to: 4 µg/ml antimicrobial peptide LL-37 for 30 min, 100 µg/ml ampicillin treatment for 30 min, competence induction with 0.4 µg/ml competence-stimulating peptide (CSP) for 12 min, sodium-chloride stress (0.5 M) for 30 min and heat-stress for 30 min at 42°C). Asteriks denote a fragment potentially corresponding to the ∼170 nt F5 RNA identified by Mann *et al*. [13]. (**D**) Northern blot analysis shows a temperature-dependent generation of F5 RNA fragments. (**E**) The heat stress-induced F5 RNA fragment represents a processed transcript, as demonstrated by 5′ RACE analysis using Cap-Clip™ Acid Pyrophosphatase (CC-AP). Total RNA from heat-stressed D39 WT cultures was either treated with CC-AP to remove 5′ caps or left untreated. RNA samples were then ligated to an RNA adapter, reverse transcribed, and amplified using adapter-specific and F5-specific primers. The resulting products were resolved on an agarose gel to assess transcript processing. Asterisks indicate the fragment that was purified and sequenced. (**F**) The 5′-end of the heat stress generated F5 RNA is located exactly 20 nt downstream the *pspA* stop codon, matching the genetic coordinates identified by Mann *et al*. (**G**) Growth experiment with D39 WT and Δ*3UTR* shows no effect of *pspA*-3′ UTR on growth management at different temperatures. Each data point represents mean of three biological replicates, with standard deviations as error bars.

We first evaluated the genomic context of the virulence-associated F5 sRNA. Based on the genomic coordinates from the study by Mann *et al*., the 5′-end of F5 is positioned 21 bp downstream of the *pspA* (SP_0117) stop codon in TIGR4, with the 3′-end being located 170 bp further downstream (Fig. 1A). The genomic sequence of F5 is highly conserved between multiple strains of pneumococcus, including TIGR4 and the D39 strain used in this study (Fig. 1A and Appendix Fig. S1). No rho-independent transcriptional terminator was predicted to be located between the *pspA* stop codon and F5. Since *S. pneumoniae* lacks the Rho-factor, the sequence analysis suggests that the *pspA* mRNA and F5 share the same transcriptional terminator, indicating that F5 could be an RNA species derived from the 185 nucleotides (nt) long *pspA* 3′-UTR. Interestingly, a 3′ UTR length of 185 nt is substantially longer than the majority of predicted 3′ UTRs in pneumococcus (>60% of 3′ UTRs at < 75 nt) (Fig. 1B), further indicating that this region could contain a functional RNA element in addition to a rho-independent transcriptional terminator.

F5 sRNA transcript levels were analyzed by northern blot analysis with pneumococcal RNA from different growth states (OD_600_ 0.1, 0.4, and 1.0) and relevant stress conditions (including antimicrobial peptide stress, beta-lactam antibiotic stress, competence induction, osmotic stress, and heat stress). At standard growth conditions, and several of the stress-related conditions, we observed relative weak bands exceeding 100 nt in size (Fig. 1C, Appendix Fig. S2). Notably, we detected a strong induction under heat stress condition, as validated by transcript level analysis at 30°C, 37°C, 40°C, and 42°C (Fig. 1D). The presence of several bands of varying sizes under heat stress conditions suggests the generation of processed fragments from an upstream RNA transcript, supporting our initial hypothesis that F5 is co-transcribed with the *pspA* mRNA. One prominently induced band, marked with an asterisk, potentially corresponds to the F5 sRNA identified by Mann *et al*. To validate this and to determine whether this F5 RNA fragment is directly transcribed from its own promoter or arises from processing of the *pspA* mRNA, we conducted a 5′ RACE analysis. This analysis revealed that the heat-stress-induced F5 RNA fragment was a processed transcript, as the treatment with the Cap-Clip™ Acid Pyrophosphatase had no clear effect on adapter ligation and reverse transcription (Fig. 1E). Sequencing of the cDNA fragment from the 5′ RACE experiment revealed that 5′-end of the RNA fragment was located 20 bp downstream of the *pspA* stop codon (Fig. 1F, Appendix Fig. S3), consistent with the genetic coordinates of F5 identified by Mann *et al*. Notably, cleavage occurred immediately downstream of a guanosine, upstream of a hairpin stem, a feature previously associated with RNase Y–mediated processing in Gram–positive bacteria [28, 29]. Together, these data demonstrate that F5 is a *pspA* mRNA 3′ UTR-derived RNA fragment.

### The protein chaperone ClpL is a regulatory target of the *pspA*-3′ UTR

To investigate the potential role of the *pspA*-3′ UTR-derived RNA fragment in pneumococcal gene regulation, we constructed a deletion mutant (Δ*3UTR*) lacking the entire F5/3′ UTR region, except for the sequence encoding the rho-independent terminator, which was retained to avoid disrupting transcription termination. The temperature-dependent processing of the *pspA*-3′ UTR suggested a possible role in growth at elevated temperatures. However, no statistically significant temperature-dependent growth effects were observed at higher temperatures (Fig. 1G, Supplementary Table S3), while only a modest but statistically significant difference was detected at 30°C.

**Figure 2.**
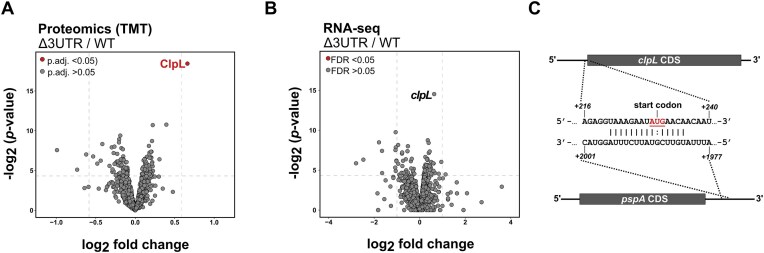
Quantitative proteomics identifies ClpL as a regulatory target of the *pspA*-3′ UTR. (**A**) Quantitative mass spectrometry shows significant upregulation of the protein chaperone ClpL in the Δ*3UTR* strain compared to the WT, as visualized in a volcano plot. Samples were harvested from cultures grown in C + Y medium at 40°C to OD_600_ 0.5. Significantly (p.adj. <0.05) and non-significantly (p.adj. >0.05) regulated proteins are depicted by red and grey dots, respectively. (**B**) RNA-sequencing of RNA from same cultures reveals a minor, non-significant (FDR > 0.05) increase of the *clpL* mRNA levels in the Δ*3UTR* strain. (**C**) Base-pairing between the *clpL*- and *pspA* mRNAs, as predicted by RNA-RNA target prediction tools, including targetRNA2, CopraRNA, and IntaRNA [33, 34].

To globally identify phenotypic changes associated with deletion of *pspA-*3′ UTR, a quantitative proteomics experiment was conducted. The D39 wild-type (WT) and Δ*3UTR* strains were grown in C + Y media to an OD_600_ 0.5 at 40°C before being harvested for quantitation by mass spectrometry. RNA from the same cultures was also harvested and sequenced by Illumina RNA-sequencing (RNA-seq). The TMT proteomics experiment identified 1217 quantifiable proteins based on at least 2 unique peptides, corresponding to ≈ 64% of the pneumococcal proteome, which is comparable to findings from previous quantitative proteomics studies in pneumococcus [30, 31, 32]. Limited effect on the proteome and transcriptome was observed between the WT and Δ*3UTR* strain. Only a single protein was significantly upregulated (∼1.6-fold upregulated, p.adj. < 1E-10) in the Δ*3UTR* strain compared to the WT. This was the Caseinolytic protease L (ClpL) (Fig. 2A), a protein chaperone and member of the heat shock protein 100 (Hsp100) family. The corresponding *clpL* mRNA levels were also slightly elevated in the Δ*3UTR* strain compared to the WT but not significantly (FDR > 0.05) (Fig. 2B). Interestingly, *clpL* being a potential regulatory target of the *pspA*-3′ UTR was consistent with predictions by target prediction tools (e.g. IntaRNA, CopraRNA and TargetRNA2 [33, 34]), which all strongly predicted the *clpL* mRNA as a potential base pairing target of the *pspA*-3′ UTR, specifically a region located near a hairpin loop structure (Fig. 2C, Appendix Fig. S4). IntaRNA predictions using the full *pspA* and *clpL* mRNAs also identified two potentially stronger interaction sites; however, these were located within the coding regions of both transcripts (Supplementary Table S4). Interestingly, when the *pspA*-3 ′ UTR was ectopically overexpressed from the chromosomal expression platform (CEP) site in a Δ*3UTR* background (Δ*3UTR* CEP:*3UTR*), no downregulation of ClpL expression was observed by quantitative proteomics (Appendix Fig. S5). This suggested that the processed F5 RNA fragment is not involved in ClpL regulation and may represent a non-functional RNA fragment.

### Full-length *pspA mRNA* post-transcriptionally regulates ClpL expression

**Figure 3.**
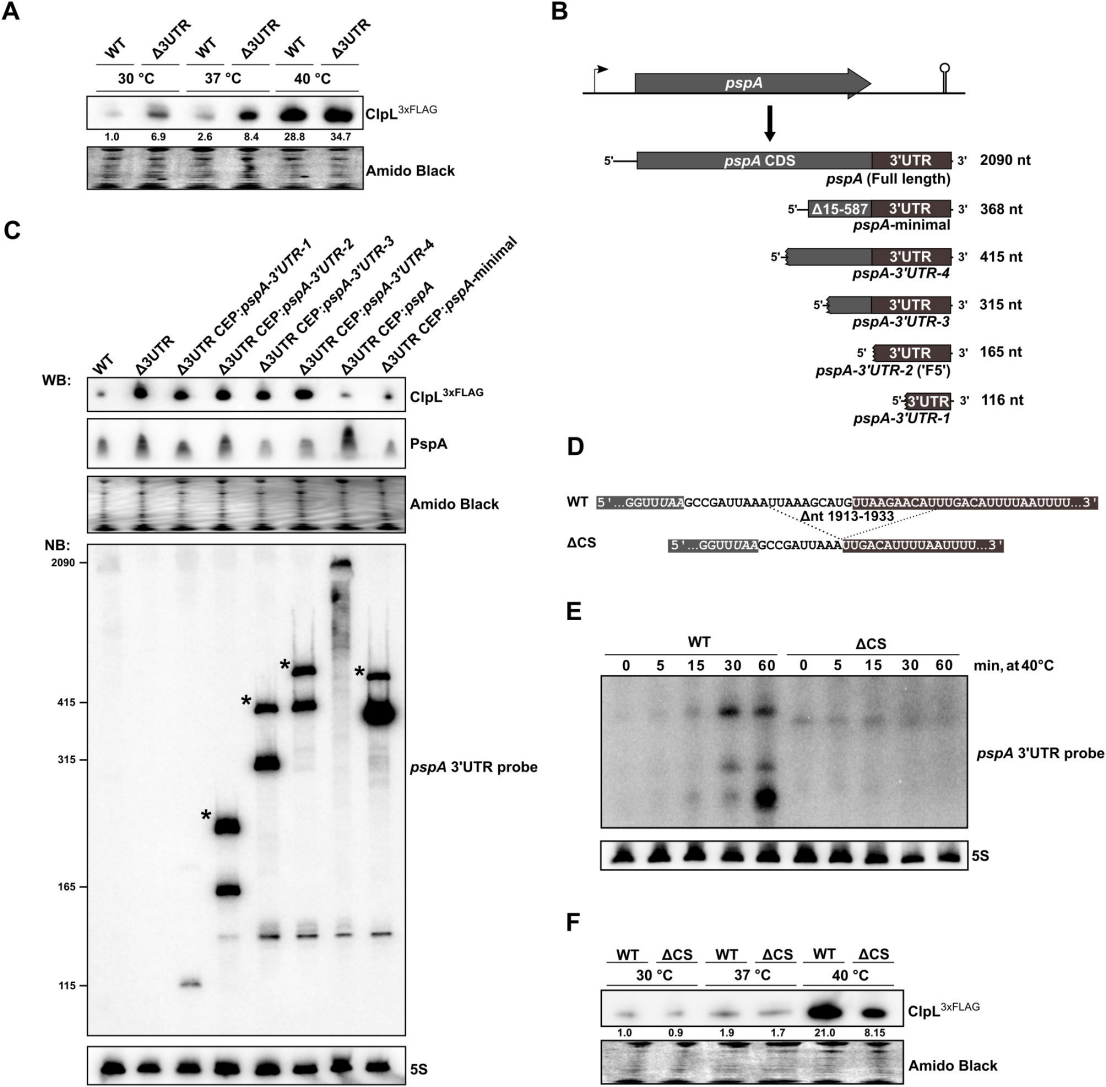
Temperature-dependent regulation of ClpL expression by full-length *pspA* mRNA. (**A**) Protein expression of ClpL^3xFLAG^ in the WT and Δ*3UTR* strains at 30°C, 37°C, and 40°C was evaluated by western blot analysis using anti-FLAG antibodies. Total protein was assessed by amido black staining. Strains listed here contain a *clpL-3xflag* fusion (WT: WT *clpL-3xFLAG*, Δ*3UTR*: Δ*3UTR clpL-3xflag*). Values represent quantification of band intensities relative to WT levels at 30°C. (**B**) Different *pspA* transcript variants were ectopically overexpressed from the chromosomal expression platform (CEP) site in a D39 Δ*3UTR* background. These included the full-length *pspA* mRNA, a *pspA* minimal variant (harboring an in-frame deletion of codon 15–587), and four 3′-truncation variants, including one corresponding to the F5 RNA (3′ UTR-2). (**C**) Protein expression of PspA and ClpL^3xFLAG^ in the specified strains at 30°C was evaluated by western blot analysis, while RNA expression of the *pspA* variants from the same cultures was evaluated by northern blot analysis using the *pspA*-3′ UTR probe, which partially targets the region deleted in the Δ*3UTR* mutant and partially targets the terminator stem. Strains listed here contain a *clpL-3xflag* fusion (WT: WT *clpL-3xFLAG*, Δ*3UTR*: Δ*3UTR clpL-3xflag*). (**D**) To evaluate the effect of temperature-dependent processing of the *pspA*-3′ UTR, a cleavage site mutant was constructed (ΔCS) containing a deletion of ± 10 bp on each side of the identified F5 5′-end site. Dark-grey color indicates the F5 region, and light-grey indicate the *pspA* CDS region. (**E**) Northern blot analysis reveals no *pspA*-3′ UTR cleavage in the ΔCS strain at 40°C. (**F**) Western blot analysis of ClpL^3xFLAG^ expression in the ΔCS strain at different temperatures (30°C, 37°C, and 40°C). Values represent quantification of band intensities relative to WT levels at 30°C.

As processing of the *pspA* mRNA increased at higher temperatures, we speculated that *pspA*-3′ UTR-mediated ClpL regulation is temperature-dependent. Western blot analysis was performed using a *clpL-3xflag* and Δ*3UTR clpL-3xflag* strain to measure ClpL levels at 30°C, 37°C, and 40°C. In both WT and Δ*3UTR* backgrounds, the levels of ClpL increased at 40°C, consistent with known regulation by the heat stress-responsive transcriptional regulator CtsR [35, 36]. Minimal differences in ClpL levels were observed at this temperature, with a more pronounced *pspA*-3′ UTR-mediated repression evident at the lower temperatures (approximately 6.9- and 3.2-fold change at 30°C and 37°C, respectively) (Fig. 3A). The increased regulatory activity at lower temperatures may help explain the modest ClpL upregulation observed in the proteomic dataset (Fig. 2A) generated at 40°C.

As *pspA*-3′ UTR-mediated repression of ClpL expression exhibited its strongest effect at lower temperatures, and the overexpression of the *pspA*-3′ UTR-derived RNA fragment did not result in ClpL downregulation, it became apparent that the heat stress-generated *pspA*-3′ UTR RNA element lacked functional regulatory properties. To identify the essential component of the full-length *pspA* mRNA required for repression of ClpL expression, we examined the overexpression of different *pspA* variants by western blot analysis at 30°C (Fig. 3B and C). These variants encompassed different 3′-end truncations, the full-length *pspA* mRNA and a designated *pspA*-minimal variant. The latter variant harbors an in-frame deletion spanning from codon 15–587 of *pspA*, while maintaining the 5′- and 3′-end of the full-length mRNA intact. No repression of ClpL expression was observed by any of the 3′-end truncations, including the one corresponding to F5 (*3′ UTR1-4*). However, when overexpressing either the full-length *pspA* mRNA or the *pspA*-minimal variant, repression of ClpL expression was observed. A northern blot analysis confirmed that the different variants were indeed overexpressed. However, it was noted that for each of the overexpressed transcripts, an extra band (approximately 100 nt longer) was also expressed (marked with an asterisk), seemingly originating from transcriptional read-through caused by incomplete *pspA* transcription termination (Appendix Fig. S6). We also observed that the *3′ UTR1* variant was overexpressed at lower levels compared to the other constructs, likely due to reduced RNA stability. This variant lacks one arm of the long stem-loop structure (Appendix Fig. S4), which may compromise its ability to resist degradation by RNases. In summary, these results indicate that it is the full-length *pspA* mRNA that regulates ClpL expression, and that repression is not caused by overexpressing of the PspA protein but relies on elements in the *pspA* 3′ UTR, possibly in cooperation with elements of the *pspA* 5′-end. In fact, IntaRNA predictions using the *pspA*-minimal construct as query did identify a possible interaction between a region in the *pspA* mRNA 5′-end and the *clpL* 5′ UTR. However, deletion of this region did not upregulate expression of ClpL (Appendix Fig. S7).

Given the high levels of *pspA* mRNA at 40°C (Appendix Fig. S8), we sought to understand why the Δ*3UTR* mutant showed only modest or no effect on ClpL levels at 40°C compared to 30°C and 37°C (Fig. 3A). We hypothesized that the increased processing of *pspA* mRNA and release of the F5 RNA element observed in Fig. 1D might contribute to the relief of ClpL repression at 40°C. To explore this further, we constructed a deletion mutant of the base pairs located in the vicinity of the *pspA* mRNA cleavage site that generates the F5 RNA fragment (± 10 bp) (Δ*CS*) (Fig. 3D), as identified by 5′ RACE (Appendix Fig. S3). This mutant did not exhibit temperature-dependent processing of the *pspA*-3′ UTR (Fig. 3E) and it showed higher repression of ClpL expression at 40°C (Fig. 3F). Additionally, mutation of the cleavage site led to increased *pspA* mRNA levels (Appendix Fig. S9), suggesting that the heat-induced cleavage destabilizes the transcript. Secondary structure predictions of the *pspA*-3′UTR region indicate that elevated temperatures reduce intramolecular base-pairing around the cleavage site, resulting in partial unfolding of local stem-loop structures (Appendix Fig. S4). This structural relaxation may enhance accessibility for RNase recognition and cleavage, consistent with the observed temperature-dependent processing. Together, our data suggests that increased processing of the WT *pspA*-3′ UTR at > 40°C relieves repression of ClpL by the *pspA* mRNA. This mechanism may help to avoid counteracting CtsR-dependent heat stress induction of ClpL expression.

### Regulation of ClpL expression by *pspA* is indirect and co-factor-dependent

**Figure 4.**
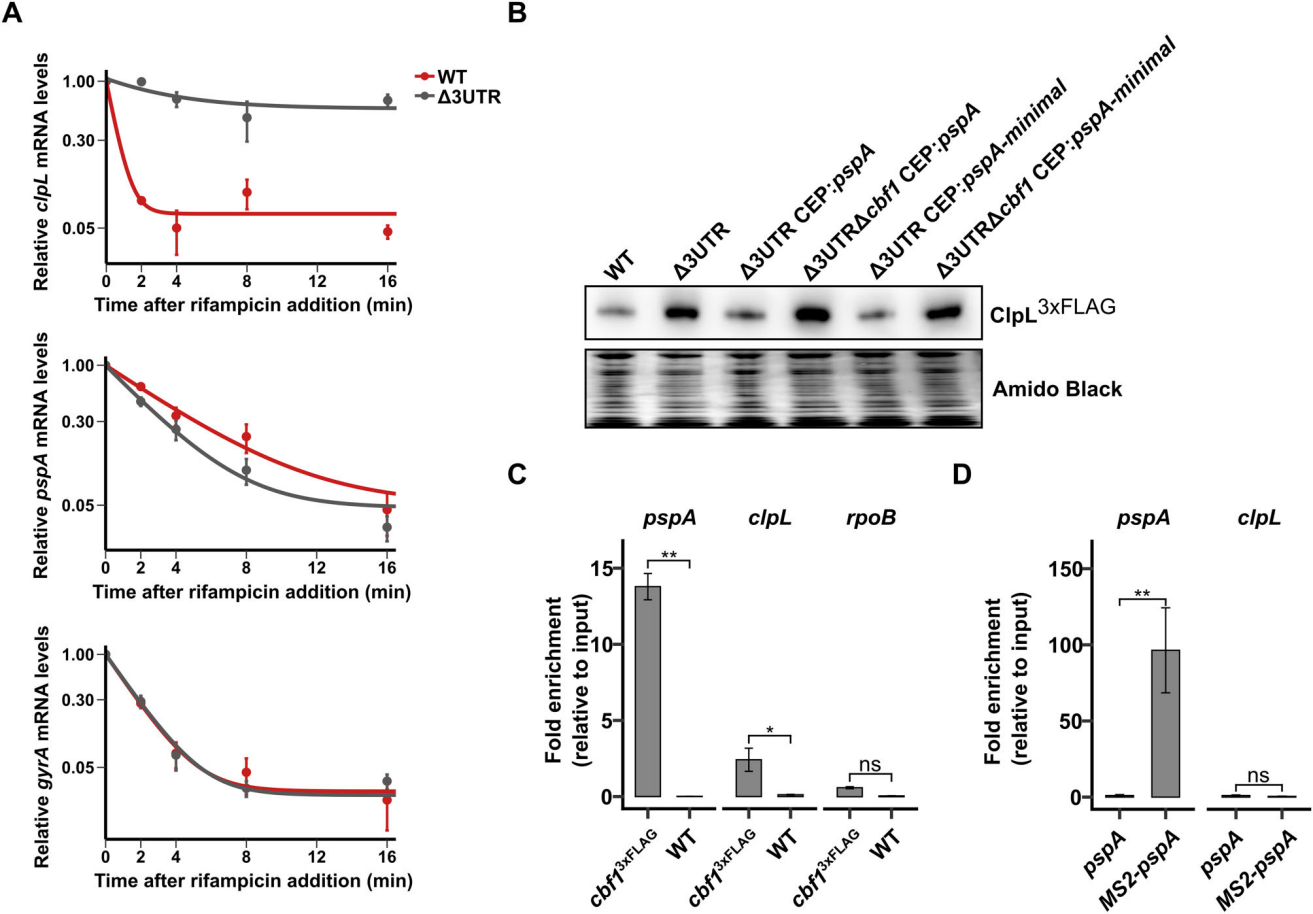
*pspA*-3′ UTR-mediated post-transcriptional regulation of *clpL* mRNA is mediated by Cbf1. (**A**) The stability of the *clpL, pspA, and gyrA (control)* mRNAs was evaluated by a rifampicin assay, in the WT and *Δ3UTR* strain at 30°C (*n* = 3). RT-qPCR was used to quantify transcript levels after 0, 2, 4, 8, and 16 min of rifampicin treatment. Graphs depicts mean transcript relative to t = 0 min, with error bars representing standard deviations, and an exponential decay function fitted to the data. (**B**) Repression of ClpL^3xFLAG^ expression by full-length *pspA* mRNA- or *pspA*-minimal overexpression was abrogated by introducing a Δ*cbf1* mutation. (**C**) A Cbf1^3xFLAG^ pull down assay displayed strong enrichment of the *pspA* mRNA, and weak enrichment of the *clpL* mRNA relative to the input in comparison to pull downs performed with untagged D39 WT. Quantification was performed by qPCR. As a non-relevant control, the *rpoB* mRNA was included. Statistical analysis was performed using two-way ANOVA followed by Tukey’s HSD post-hoc test to assess pairwise differences in fold enrichments (**: *P* < 0.01, *: *P* < 0.05, ns: *P* > 0.05). (**D**) Co–enrichment of *clpL* mRNA was not observed following MS2 affinity purification using MS2–tagged full–length *pspA* mRNA overexpressed from the CEP site (Δ*pspA* CEP: 5′–*MS2–pspA* (*MS2-pspA*)). Cultures used for pull–down were grown at 30°C to an OD₆₀₀ of 0.6. A strain expressing untagged *pspA* mRNA (Δ*pspA* CEP:*pspA*, (*pspA*)) served as a control. Statistical analysis was performed using two–way ANOVA followed by Tukey’s HSD post–hoc test to assess pairwise differences in fold enrichment (** p < 0.01, * p < 0.05, ns p > 0.05).

Rifampicin assays at 30°C revealed that increased levels of ClpL at lower temperatures in the Δ*3UTR* mutant could be explained by increased *clpL* mRNA stability, supporting a potential post-transcriptional mechanism (Fig. 4A). This mechanism may involve base pairing, as indicated by the predicted target interactions (Fig. 2C). Based on gel shift assays we were not able to detect any interaction between *in vitro* synthesized *pspA-* and *clpL* RNA constructs. The constructs used included a 287-nucleotide fragment from the 5′ end of the *clpL* mRNA, encompassing its 5′ UTR and the coding sequence for the first 19 codons, combined with either the *pspA* 3′ UTR transcript or the regulatory *pspA*-minimal RNA construct. No shift was observed under any of the tested conditions, except for a very weak and insignificant shift (Appendix Fig. S10). Instead, we suspected that a co-factor such as an RNA-binding protein (RBP) was involved in mediating the RNA interactions *in vivo*. However, to date, no functional homologs of prototypical RBPs such as Hfq and ProQ have been identified in *S. pneumoniae*. Recently, Hör *et al*. performed gradient profiling by sequencing (Grad-seq) to discover novel RNA-binding proteins in pneumococcus [37]. They discovered that the 3′-5′-exonuclease Cbf1 is involved in sRNA stabilization by 3′-end trimming. They additionally identified multiple ncRNAs as potential interaction partners of Cbf1, including F5. Based on this, we speculated that Cbf1 is involved in *pspA*-mediated ClpL regulation, possibly by facilitating the RNA-RNA interaction, directly or indirectly. The effect of Cbf1 on *pspA*-mediated regulation was investigated by western blot analysis using a *cbf1* deletion mutant (Δ*cbf1)*. Indeed, repression of ClpL expression by full-length *pspA* mRNA- or *pspA*-minimal overexpression was abrogated by introducing the Δ*cbf1* mutation (Fig. 4B). Also, enrichment of the *pspA* mRNA was observed in a Cbf1-3xFLAG pull down experiment, further validating the *pspA* mRNA as a direct interaction partner of Cbf1 (Fig. 4C). Weak enrichment of *clpL* mRNA was observed in the pull–down experiment, which may reflect either co–enrichment mediated by interaction with the *pspA* mRNA or a direct interaction between *clpL* mRNA and Cbf1. Adding purified Cbf1-3xFLAG to *in vitro* gel shift assays did not stimulate an interaction between the *in vitro* transcribed *clpL-* and *pspA* transcripts (Appendix Fig. S10), either suggesting that the role of Cbf1 in *pspA*-mediated ClpL regulation does not involve facilitating the RNA-RNA interaction, or that additional factors are involved *in vivo*.

Instead, to identify a potential *pspA*–*clpL* mRNA interaction *in vivo*, MS2 affinity purification was performed using a strain overexpressing a 5′ MS2–tagged *pspA* mRNA and an untagged control. While the tagged *pspA* transcript was efficiently enriched, no co-enrichment of *clpL* mRNA was detected (Fig. 4D). This finding is inconsistent with a stable *pspA*–*clpL* RNA–RNA interaction and suggests indirect regulation or a highly transient interaction.

Since Cbf1 is required for pspA–dependent regulation of ClpL, we next tested whether this effect could be explained by altered *pspA* mRNA stability mediated by Cbf1–dependent 3′–end trimming, which was previously shown for the csRNAs (Hör *et al*., 2020a). While evidence of *pspA*-3′ UTR trimming was observed in a Cbf1 incubation assay (Appendix Fig. S11A), *cbf1* deletion had only a minor negative impact on *pspA* mRNA stability (Appendix Fig. S11B), which is already quite low in a WT background (t½ ∼ 1.5 min). Furthermore, steady-state levels were either unchanged or increased in the Δ*cbf1* strain, depending on the temperature, indicating that Cbf1 is not involved in stabilizing the *pspA* mRNA transcript (Appendeix Fig. S11C).

### The *pspA-*3′ UTR plays a role in inhibition of complement deposition

**Figure 5.**
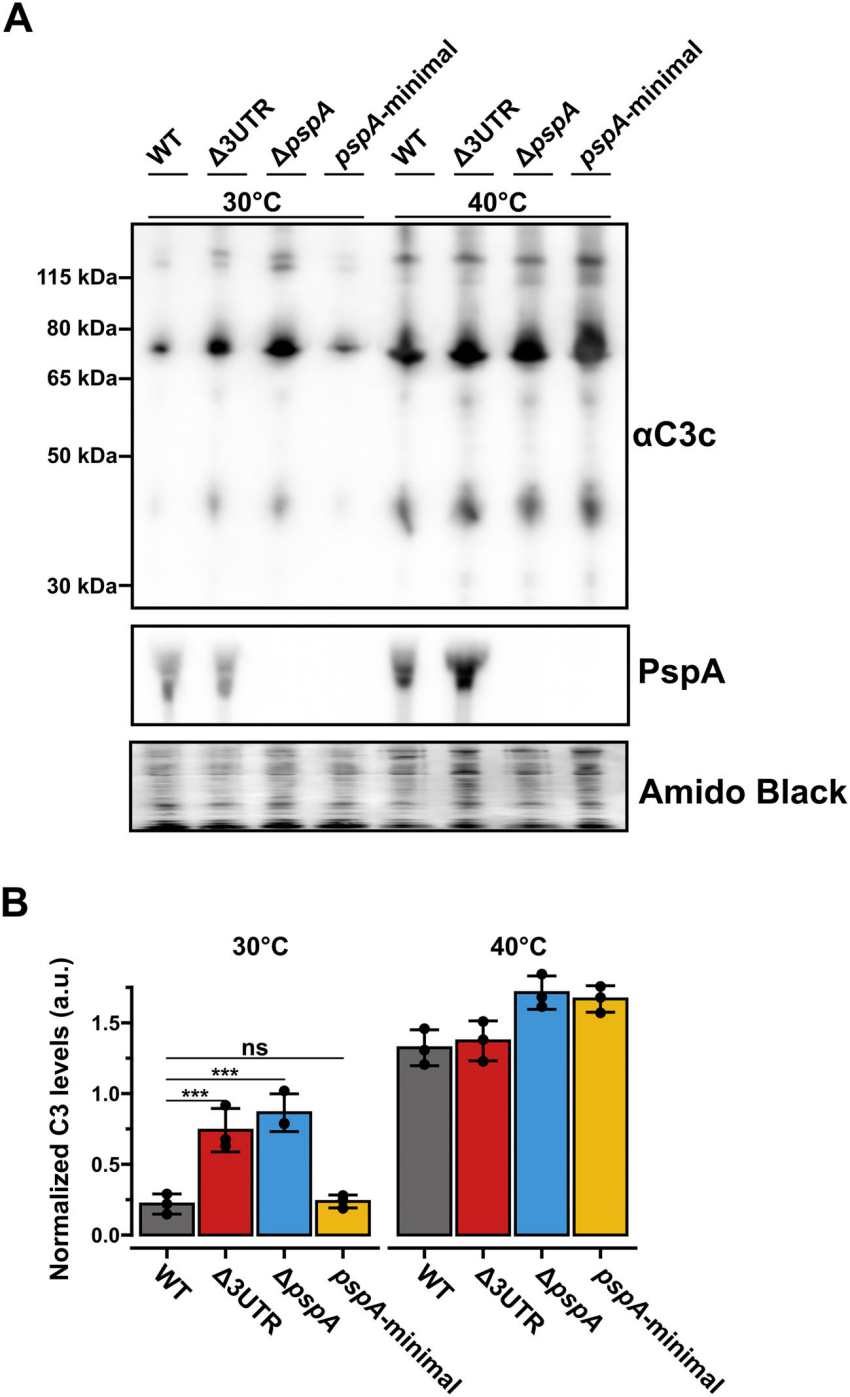
The *pspA*-3′ UTR affects complement C3 deposition at lower temperatures. (**A**) Deposition of human complement C3 on the D39 WT, Δ*3UTR*, Δ*pspA* and *pspA*-minimal strains was evaluated at 30°C and 40°C by western blot analysis using anti-C3c antibodies, which also recognize surface-bound C3b. The blot shows multiple C3-derived fragments, including prominent bands at approximately 115 kDa and 75 kDa, which are consistent with the expected sizes of the α-chain and β-chain, respectively. Expression of PspA was validated using polyclonal anti-PspA antibodies. (**B**) Complement C3 deposition was compared between strains at 30°C and 40°C. Band intensities of the ∼115 kDa and ∼75 kDa species were quantified to evaluate relative C3 deposition. Data represent means of three biological replicates with error bars indicating standard deviations. Statistical significance was assessed using one-way ANOVA followed by Tukey’s post hoc test. Asterisks denote levels of statistical significance (ns: *P* > 0.05, *: *P* < 0.001).

With PspA playing an important role in inhibiting surface deposition of complement C3, we aimed to investigate the effect of the regulatory *pspA*-3′ UTR on complement C3 deposition using human serum. Previous studies examining the impact of PspA on complement C3 deposition have predominantly utilized complete *pspA* knockout strains, which also removes the 3′ UTR [18, 20, 38]. As a result, these studies do not differentiate between the effects caused by the PspA protein and by the regulatory *pspA-*3′ UTR. In our experimental setup, we analyzed complement deposition in the WT (*pspA-*3′ UTR-positive, PspA-positive), Δ*3UTR* (*pspA-*3′ UTR-negative, PspA-positive), Δ*pspA* (*pspA-*3′ UTR-negative, PspA-negative), and the *pspA-*minimal (*pspA-*3′ UTR-positive, PspA-negative) strains. Given the temperature-dependent regulation by the *pspA-*3′ UTR, we examined complement deposition at 30°C and 40°C. The strains were grown in C + Y medium at these two temperatures until reaching an OD_600_ 0.05, after which 20% human serum was added to each culture, followed by a 2-h incubation. Western blot analysis with anti-C3c antibodies was performed out to measure C3 deposition. The results show a significant increase in complement C3 deposition in the Δ*3UTR* and Δ*pspA* strains at 30°C (*p* < 0.001), while no increase was observed for the *pspA-*minimal strain (Fig. 5A and B). This suggests that the *pspA*-3′ UTR region plays a more significant role in inhibiting complement deposition at 30°C compared to the PspA protein. In contrast, only small differences were observed between strains at 40°C, with a slight increase in C3 deposition in the PspA-negative strains (Δ*pspA* and *pspA-*minimal) at 40°C. This indicates that while the *pspA*-3′ UTR might be important for inhibition of C3 deposition at 30°C, it has no such effect at higher temperatures, consistent with its strongest regulatory activity occurring at 30°C.

We hypothesized that the *pspA*-3′ UTR could influence complement deposition by modulating ClpL’s chaperone activity on PspA. Interestingly, ClpL also localizes to the cell wall during heat stress, where PspA is also found (Appendix Fig. S12A). However, preliminary protein stability assays showed no clear effect of *clpL* deletion on PspA stability (Appendix Fig. S12B).

## Discussion

The pneumococcal virulence factor PspA has been described as a “jack of all trades,” with roles in complement inhibition, antimicrobial peptide neutralization, host cell adherence, and exploitation of host metabolism [24]. Our study extends this functional versatility by uncovering a novel post-transcriptional role of the *pspA* mRNA, mediated through its 3′ UTR, which contributes to complement inhibition in a temperature-dependent manner.

Bacterial 3′ UTRs are increasingly recognized as key hubs for post-transcriptional regulation, acting as reservoirs for *trans*-acting sRNAs that may be generated through mRNA processing (e.g. PlyT) or transcribed from internal promoters [39, 40]. Although processing within the *pspA* 3′ UTR was observed, regulatory activity was not associated with processed RNA species but instead required the intact *pspA* mRNA. Such dual–function mRNAs, which combine a coding sequence with an unprocessed riboregulatory element, have been documented previously [41]. However, apart from *hly* of *L. monocytogenes*, most of these are typically short mRNAs (<500 nt) encoding short polypeptides (e.g. SgrS and RNAIII) [42–45]. The *pspA* mRNA represents a novel addition to this class of long dual-function mRNAs, suggesting that such mRNAs may be more widespread. Given that ∼25% of 3′ UTRs in *S. pneumoniae* exceed 100 nt (Fig. 1B), it is likely that many of these contain riboregulatory elements.

Our data demonstrate that *pspA* mRNA regulates ClpL expression post–transcriptionally, but this regulation does not appear to rely on direct RNA–RNA base pairing. No interaction between *pspA* and the *clpL* 5′ region was detected in *in vitro* gel–shift assays, nor did *in vivo* MS2 affinity purification provide evidence for a stable *pspA*–*clpL* RNA interaction. Furthermore, targeted deletion of nucleotides predicted to be involved in base pairing did not affect regulation (unpublished observations). Instead, regulation was found to require the full–length *pspA* mRNA, in a process that is dependent on Cbf1. Although Cbf1 is required for *pspA*–mediated repression of ClpL, the data do not allow conclusions about how Cbf1 modulates this regulatory process. Together, these findings argue for an indirect, co–factor–dependent mechanism in which *pspA* does not act through canonical antisense pairing. Whether this indirect mode of regulation involves recruitment or sequestration of RNA–binding proteins such as Cbf1, or other *clpL* mRNA–interacting factors that modulate its accessibility or translation, remains to be resolved.

Despite the absence of direct RNA–RNA interaction, the specific effect of *pspA* 3′ UTR deletion on ClpL expression suggests a functionally meaningful relationship rather than a nonspecific regulatory consequence. For other dual–function RNAs, the regulatory RNA and its encoded protein often act within the same or related cellular pathways [41], raising the possibility that *pspA*-mediated regulation of *clpL* reflects a downstream functional linkage. In *L. monocytogenes*, for example, the dual-function *hly* mRNA regulates expression of *prsA2*, encoding a chaperone required for folding of secreted proteins, including LLO encoded by *hly* itself [42]. Similarly, we speculated that PspA could be a substrate for the protein chaperone ClpL. Interestingly, previous studies and our own data suggest that ClpL localizes to the cell wall during heat stress [46] (Appendix Fig. S12A), where PspA is also located. Preliminary data from this study reveal that ClpL does not impact the stability of the PspA protein, indicating that PspA is not a substrate for ClpL, and that a functional link should instead be found between PspA and the downstream effectors of ClpL. Notably, ClpL has been linked to virulence by modulating adhesion to host cells and penicillin susceptibility through modulation of the cell wall biosynthesis enzyme penicillin-binding protein 2X (Pbp2X), suggesting a role in cell surface functions potentially relevant to PspA activity [46–48].

The most important aspect of PspA-mediated virulence has been credited to its ability to interfere with the classical pathway of complement deposition, which plays a key role in host defense against the pneumococcus [49]. Despite evidence suggesting a direct role of PspA in blocking the binding of C-reactive protein (CRP) to phosphocholine in the pneumococcal cell wall, and thereby inhibiting classical pathway activation and C3 deposition [17], this direct role in inhibition of complement deposition has not been explored further in literature from the last decade. Our study highlights the dual function of the *pspA* mRNA and underscores the importance of distinguishing effects on complement deposition caused by the PspA protein and by the riboregulatory function of the *pspA* mRNA. Our data suggest that the regulatory *pspA-*3′ UTR plays a larger role in C3 deposition compared to the PspA protein, although this effect is observed at lower temperatures. This may not be physiologically relevant, as complement is primarily active at 37°C or higher during fever. Therefore, the differences in complement deposition at 30°C could reflect an indirect phenotype arising from other effects of *pspA*–mediated regulation. Alternatively, the apparent temperature dependence may relate to variations in growth rate rather than temperature per se. Growth rate could be a critical factor influencing *pspA*-mediated regulation, particularly in the bloodstream, where other factors than temperature may influence growth. Cell division has already been established as a vulnerable phase for the pneumococcus, when it comes to complement deposition, as the cell wall near the division septum is a major target for C3 deposition [50, 51]. This is also evident in our data that shows increased C3 deposition at the temperature with the higher growth rate, 40°C (Figs 1G and 5).

Our findings provide novel insights into the riboregulatory function of *pspA* mRNA, which encodes one of the most prominent virulence factors of pneumococcus. This dual role underscores the complex mechanisms through which *pspA* contributes to pneumococcal pathogenesis, including its modulation of complement deposition. The impact of *pspA*-mediated ClpL regulation on complement deposition warrants further investigation. As discussed, ClpL’s effects on cell surface functions could be a contributing factor, considering the cell wall is a primary target for complement deposition [50, 51]. Given PspA’s potential as a vaccine antigen [52–54, 55], further exploration of the riboregulatory functions of the *pspA* mRNA in modulating immune responses will be essential for a deeper understanding of its role in immune evasion and complement inhibition.

## Supplementary Material

gkag481_Supplemental_Files

## Data Availability

The RNA-sequencing data discussed in this publication have been deposited in NCBI’s Gene Expression Omnibus [56] and are accessible through GEO Series accession number GSE225655 (https://www.ncbi.nlm.nih.gov/geo/query/acc.cgi?acc=GSE225655). The mass spectrometry proteomics data have been deposited to the ProteomeXchange Consortium (http://proteomecentral.proteomexchange.org) via the PRIDE partner repository [57] with the dataset identifier PXD040356.

## References

[1] Waters LS, Storz G. Regulatory RNAs in bacteria. Cell. 2009;136:615–28. 10.1016/j.cell.2009.01.043. 19239884 PMC3132550

[2] Jørgensen MG, Pettersen JS, Kallipolitis BH. sRNA-mediated control in bacteria: an increasing diversity of regulatory mechanisms. Biochim Biophys Acta Gene Regulatory Mech. 2020;1863:194504. 10.1016/j.bbagrm.2020.194504. 32061884

[3] Eichner H, Karlsson J, Loh E. The emerging role of bacterial regulatory RNAs in disease. Trends Microbiol. 2022;30:959–72. 10.1016/j.tim.2022.03.007. 35379550

[4] Hör J, Matera G, Vogel J et al. Trans-acting small RNAs and their effects on gene expression in *Escherichia coli* and *Salmonella enterica*. EcoSal Plus. 2020;9. 10.1128/ecosalplus.esp-0030-2019. PMC7112153 32213244

[5] Chakravarty S, Massé E. RNA-dependent regulation of virulence in pathogenic bacteria. Front Cell Infect Microbiol. 2019;9:337–. 10.3389/fcimb.2019.00337. 31649894 PMC6794450

[6] Subramanian K, Henriques-Normark B, Normark S. Emerging concepts in the pathogenesis of the Streptococcus pneumoniae: from nasopharyngeal colonizer to intracellular pathogen. Cell Microbiol. 2019;21:e13077–. 10.1111/cmi.13077. 31251447 PMC6899785

[7] Weiser JN, Ferreira DM, Paton JC. Streptococcus pneumoniae: transmission, colonization and invasion. Nat Rev Micro. 2018;16:355–67. 10.1038/s41579-018-0001-8. PMC5949087 29599457

[8] Aprianto R, Slager J, Holsappel S et al. Time-resolved dual RNA-seq reveals extensive rewiring of lung epithelial and pneumococcal transcriptomes during early infection. Genome Biol. 2016;17:198. 10.1186/s13059-016-1054-5. 27678244 PMC5039909

[9] Aprianto R, Slager J, Holsappel S et al. High-resolution analysis of the pneumococcal transcriptome under a wide range of infection-relevant conditions. Nucleic Acids Res. 2018;46:9990–10006. 30165663 10.1093/nar/gky750 PMC6212715

[10] Hava DL, Camilli A. Large-scale identification of serotype 4 *Streptococcus pneumoniae* virulence factors. Mol Microbiol. 2002;45:1389–406. 12207705 PMC2788772

[11] D’Mello A, Riegler AN, Martínez E et al. An in vivo atlas of host-pathogen transcriptomes during Streptococcus pneumoniae colonization and disease. Proc Natl Acad Sci USA. 2020;117:33507–18. 10.1073/pnas.2010428117. 33318198 PMC7777036

[12] Sinha D, Zimmer K, Cameron TA et al. Redefining the small regulatory RNA transcriptome in *Streptococcus pneumoniae* serotype 2 strain D39. J Bacteriol. 2019;201:e00764–18. 10.1128/JB.00764-18. 30833353 PMC6597385

[13] Mann B, van Opijnen T, Wang J et al. Control of virulence by small RNAs in Streptococcus pneumoniae. PLoS Pathog. 2012;8:e1002788. 10.1371/journal.ppat.1002788. 22807675 PMC3395615

[14] Acebo P, Martin-Galiano AJ, Navarro S et al. Identification of 88 regulatory small RNAs in the TIGR4 strain of the human pathogen Streptococcus pneumoniae. RNA. 2012;18:530–46. 10.1261/rna.027359.111. 22274957 PMC3285940

[15] Schnorpfeil A, Kranz M, Kovács M et al. Target evaluation of the non-coding csRNAs reveals a link of the two-component regulatory system CiaRH to competence control in Streptococcus pneumoniae R6. Mol Microbiol. 2013;89:334–49. 10.1111/mmi.12277. 23710838

[16] Shen K, Miao W, Zhu L et al. A 3’UTR-derived small RNA represses pneumolysin synthesis and facilitates pneumococcal brain invasion. Commun Biol. 2024;7:1130. 10.1038/s42003-024-06845-8. 39271946 PMC11399405

[17] Mukerji R, Mirza S, Roche AM et al. Pneumococcal surface protein A inhibits complement deposition on the pneumococcal surface by competing with the binding of C-reactive protein to cell-surface phosphocholine. J Immunol. 2012;189:5327–35. 10.4049/jimmunol.1201967. 23105137 PMC3517878

[18] Ren B, Szalai AJ, Hollingshead SK et al. Effects of PspA and antibodies to PspA on activation and deposition of complement on the pneumococcal surface. Infect Immun. 2004;72:114–22. 10.1128/IAI.72.1.114-122.2004. 14688088 PMC344006

[19] Shaper M, Hollingshead SK, Benjamin WH Jr et al. PspA protects Streptococcus pneumoniae from killing by apolactoferrin, and antibody to PspA enhances killing of pneumococci by apolactoferrin [corrected]. Infect Immun. 2004;72:5031–40. 10.1128/IAI.72.9.5031-5040.2004. 15321996 PMC517438

[20] Tu AH, Fulgham RL, McCrory MA et al. Pneumococcal surface protein A inhibits complement activation by Streptococcus pneumoniae. Infect Immun. 1999;67:4720–4. 10.1128/IAI.67.9.4720-4724.1999. 10456922 PMC96800

[21] Ren B, Li J, Genschmer K et al. The absence of PspA or presence of antibody to PspA facilitates the complement-dependent phagocytosis of pneumococci *In Vitro*. Clin Vaccine Immunol. 2012;19:1574–82. 10.1128/CVI.00393-12. 22855389 PMC3485889

[22] Park SS, Gonzalez-Juarbe N, Riegler AN et al. *Streptococcus pneumoniae* binds to host GAPDH on dying lung epithelial cells worsening secondary infection following influenza. Cell Rep. 2021;35:109267. 10.1016/j.celrep.2021.109267. 34133917 PMC8265312

[23] Park SS, Gonzalez-Juarbe N, Martínez E et al. *Streptococcus pneumoniae* binds to host lactate dehydrogenase via PspA and PspC to enhance virulence. mBio. 2021;12:e00673–21. 10.1128/mBio.00673-21. 33947761 PMC8437407

[24] Lane JR, Tata M, Briles DE et al. A jack of all trades: the role of pneumococcal surface protein A in the pathogenesis of *Streptococcus pneumoniae*. Front Cell Infect Microbiol. 2022;12:826264. 10.3389/fcimb.2022.826264. 35186799 PMC8847780

[25] Sorg RA, Kuipers OP, Veening JW. Gene expression platform for synthetic biology in the human pathogen *Streptococcus pneumoniae*. ACS Synth Biol. 2015;4:228–39. 10.1021/sb500229s. 24845455

[26] Price KE, Camilli A. Pneumolysin localizes to the cell wall of *Streptococcus pneumoniae*. J Bacteriol. 2009;191:2163–8. 10.1128/JB.01489-08. 19168620 PMC2655535

[27] Heissel S, Bunkenborg J, Kristiansen MP et al. Evaluation of spectral libraries and sample preparation for DIA-LC-MS analysis of host cell proteins: a case study of a bacterially expressed recombinant biopharmaceutical protein. Protein Expression Purif. 2018;147:69–77. 10.1016/j.pep.2018.03.002. 29526817

[28] Broglia L, Lécrivain AL, Renault TT et al. An RNA-seq based comparative approach reveals the transcriptome-wide interplay between 3'-to-5' exoRNases and RNase Y. Nat Commun. 2020;11:1587. 10.1038/s41467-020-15387-6. 32221293 PMC7101322

[29] Le Scornet A, Jousselin A, Baumas K et al. Critical factors for precise and efficient RNA cleavage by RNase Y in *Staphylococcus aureus*. PLoS Genet. 2024;20:e1011349. 10.1371/journal.pgen.1011349. 39088561 PMC11321564

[30] Hirschfeld C, Gómez-Mejia A, Bartel J et al. Proteomic investigation uncovers potential targets and target sites of pneumococcal serine-threonine kinase StkP and phosphatase PhpP. Front Microbiol. 2020;10:3101. 10.3389/fmicb.2019.03101. 32117081 PMC7011611

[31] Miao X, He J, Zhang L et al. A novel iron transporter SPD_1590 in *Streptococcus pneumoniae* contributing to bacterial virulence properties. Front Microbiol. 2018;9:1624–. 10.3389/fmicb.2018.01624. 30079056 PMC6062600

[32] Shen K, Hu Q, Zhu L et al. Pn-AqpC-mediated fermentation pattern coordination with the two-component system 07 regulates host N-glycan degradation of *Streptococcus pneumoniae*. Microbiol Spectr. 2022;10:e0249622–. 10.1128/spectrum.02496-22. 36106896 PMC9603416

[33] Kery MB, Feldman M, Livny J et al. TargetRNA2: identifying targets of small regulatory RNAs in bacteria. Nucleic Acids Res. 2014;42:W124–9. 10.1093/nar/gku317. 24753424 PMC4086111

[34] Wright PR, Georg J, Mann M et al. CopraRNA and IntaRNA: predicting small RNA targets, networks and interaction domains. Nucleic Acids Res. 2014;42:W119–23. 10.1093/nar/gku359. 24838564 PMC4086077

[35] Derré I, Rapoport G, Msadek T. CtsR, a novel regulator of stress and heat shock response, controls clp and molecular chaperone gene expression in Gram-positive bacteria. Mol Microbiol. 1999;31:117–31. 9987115 10.1046/j.1365-2958.1999.01152.x

[36] Dagkessamanskaia A, Moscoso M, Hénard V et al. Interconnection of competence, stress and CiaR regulons in *Streptococcus pneumoniae*: competence triggers stationary phase autolysis of ciaR mutant cells. Mol Microbiol. 2004;51:1071–86. 10.1111/j.1365-2958.2003.03892.x. 14763981

[37] Hör J, Garriss G, Di Giorgio S et al. Grad-seq in a Gram-positive bacterium reveals exonucleolytic sRNA activation in competence control. EMBO J. 2020;39:e103852–. 32227509 10.15252/embj.2019103852 PMC7196914

[38] Haleem KS, Ali YM, Yesilkaya H et al. The pneumococcal surface proteins PspA and PspC sequester host C4-binding protein to inactivate complement C4b on the bacterial surface. Infect Immun. 2019;87:e00742–00718. 10.1128/IAI.00742-18. PMC6300637 30323030

[39] Ponath F, Hör J, Vogel J. An overview of gene regulation in bacteria by small RNAs derived from mRNA 3' ends. FEMS Microbiol Rev. 2022;46:fuac017. 10.1093/femsre/fuac017. 35388892 PMC9438474

[40] Menendez-Gil P, Toledo-Arana A. Bacterial 3′UTRs: a useful resource in post-transcriptional regulation. Front Mol Biosci. 2021;7:617633. 10.3389/fmolb.2020.617633. 33490108 PMC7821165

[41] Raina M, King A, Bianco C et al. Dual-function RNAs. Microbiol Spectr. 2018;6. 10.1128/microbiolspec.RWR-0032-2018. PMC6130917 30191807

[42] Ignatov D, Vaitkevicius K, Durand S et al. An mRNA-mRNA interaction couples expression of a virulence factor and its chaperone in listeria monocytogenes. Cell Rep. 2020;30:4027–4040.e7. 10.1016/j.celrep.2020.03.006. 32209466 PMC8722363

[43] Vanderpool CK, Balasubramanian D, Lloyd CR. Dual-function RNA regulators in bacteria. Biochimie. 2011;93:1943–9. 10.1016/j.biochi.2011.07.016. 21816203 PMC3185123

[44] Novick RP, Ross HF, Projan SJ et al. Synthesis of Staphylococcal virulence factors is controlled by a regulatory RNA molecule. EMBO J. 1993;12:3967–75. 10.1002/j.1460-2075.1993.tb06074.x. 7691599 PMC413679

[45] Wadler CS, Vanderpool CK. A dual function for a bacterial small RNA: sgrS performs base pairing-dependent regulation and encodes a functional polypeptide. Proc Natl Acad Sci USA. 2007;104:20454–9. 10.1073/pnas.0708102104. 18042713 PMC2154452

[46] Tran TD-H, Kwon H-Y, Kim E-H et al. Decrease in penicillin susceptibility due to heat shock protein ClpL in *Streptococcus pneumoniae*. Antimicrob Agents Chemother. 2011;55:2714–28. 10.1128/AAC.01383-10. 21422206 PMC3101445

[47] Tu LN, Jeong H-Y, Kwon H-Y et al. Modulation of adherence, invasion, and tumor necrosis factor alpha secretion during the early stages of infection by *Streptococcus pneumoniae* ClpL. Infect Immun. 2007;75:2996–3005. 10.1128/IAI.01716-06. 17403879 PMC1932908

[48] Nguyen CT, Le N-T, Tran TD-H et al. *Streptococcus pneumoniae* ClpL modulates adherence to A549 human lung cells through Rap1/Rac1 activation. Infect Immun. 2014;82:3802–10. 10.1128/IAI.02012-14. 24980975 PMC4187815

[49] Brown JS, Hussell T, Gilliland SM et al. The classical pathway is the dominant complement pathway required for innate immunity to *Streptococcus pneumoniae* infection in mice. Proc Natl Acad Sci USA. 2002;99:16969–74. 10.1073/pnas.012669199. 12477926 PMC139253

[50] Hummell DS, Berninger RW, Tomasz A et al. The fixation of C3b to pneumococcal cell wall polymers as a result of activation of the alternative complement pathway. J Immunol. 1981;127:1287–9. 10.4049/jimmunol.127.4.1287. 7024404

[51] Pathak A, Bergstrand J, Sender V et al. Factor H binding proteins protect division septa on encapsulated *Streptococcus pneumoniae* against complement C3b deposition and amplification. Nat Commun. 2018;9:3398. 10.1038/s41467-018-05494-w. 30139996 PMC6107515

[52] Seo SU, Kim JJ, Yang H et al. Effective protection against secondary pneumococcal pneumonia by oral vaccination with attenuated Salmonella delivering PspA antigen in mice. Vaccine. 2012;30:6816–23. 10.1016/j.vaccine.2012.09.015. 23000127

[53] Masomian M, Ahmad Z, Gew LT et al. Development of next generation *Streptococcus pneumoniae* vaccines conferring broad protection. Vaccines. 2020;8:132. 10.3390/vaccines8010132. 32192117 PMC7157650

[54] Briles DE, Hollingshead SK, King J et al. Immunization of humans with recombinant pneumococcal surface protein A (rPspA) elicits antibodies that passively protect mice from fatal infection with *Streptococcus pneumoniae* bearing heterologous PspA. J Infect Dis. 2000;182:1694–701. 10.1086/317602. 11069242

[55] Nabors GS, Braun PA, Herrmann DJ et al. Immunization of healthy adults with a single recombinant pneumococcal surface protein A (PspA) variant stimulates broadly cross-reactive antibodies to heterologous PspA molecules. Vaccine. 2000;18:1743–54. 10.1016/S0264-410X(99)00530-7. 10699322

[56] Edgar R, Domrachev M, Lash AE. Gene expression omnibus: NCBI gene expression and hybridization array data repository. Nucleic Acids Res. 2002;30:207–10. 10.1093/nar/30.1.207. 11752295 PMC99122

[57] Perez-Riverol Y, Bai J, Bandla C et al. The PRIDE database resources in 2022: a hub for mass spectrometry-based proteomics evidences. Nucleic Acids Res. 2022;50:D543–52. 10.1093/nar/gkab1038. 34723319 PMC8728295

