# Supplementary material for: 3′ UTR-mediated regulation of a protein chaperone by the pspA mRNA in Streptococcus pneumoniae
Source: Nucleic Acids Res. 2026 May 14;54(9):gkag481. doi: 10.1093/nar/gkag481 (PMC13173289; doi:10.1093/nar/gkag481)
Supplement: gkag481_Supplemental_Files [file gkag481_supplemental_files.zip › Supplementary_file_23_04_2026.docx]

**Appendix – Table of Contents**

Appendix Figure S1 - Conservation of F5 genomic region across pneumococcal species. 3

Appendix Figure S2 – F5 Northern blot with RNA ladder. 4

Appendix Figure S3 - Mapping the 5’-end of the F5 RNA fragment by 5’ RACE analysis. 5

Appendix Figure S4 – Predicted secondary structure of the pspA 3’UTR region across temperatures. 6

Appendix Figure S5 - Quantitative proteomics of D39 Δ3UTR CEP:3UTR compared to wild type. 7

Appendix Figure S6 - Read-through transcription at the CEP-site. 8

Appendix Figure S7 – Western blot analysis of ClpL expression in a *pspA*-5’ UTR deletion mutant (Δ5UTR). 9

Appendix Figure S8 - Expression of *pspA* mRNA at different temperatures. 10

Appendix Figure S9 – Northern blot analysis of *pspA* mRNA and cleavage products in wild-type (WT) and ΔCS mutant strains at 30°C and 40°C. 11

Appendix Figure S10 - Investigation of *in vitro* interaction between the *clpL*-5’ RNA and *pspA*-3’ *RNA* or *pspA* minimal constructs by gel shift assays. 12

Appendix Figure S11 – Analysis of Cbf1-mediated of *pspA*-3’-end trimming. 14

Appendix Figure S12 – Western blot analysis of PspA stability and ClpL localization. 15

Appendix Table S1 – Strain list. 16

Appendix Table S2 – Oligo list. 16

Appendix Table S3 – Generation times of D39 WT and Δ*3UTR* strains at 30, 37 and 40 °C. 17

Appendix Table S4 – Top five predicted interactions between the *pspA* and *clpL* mRNAs, as predicted by IntaRNA. 18

Appendix references 19


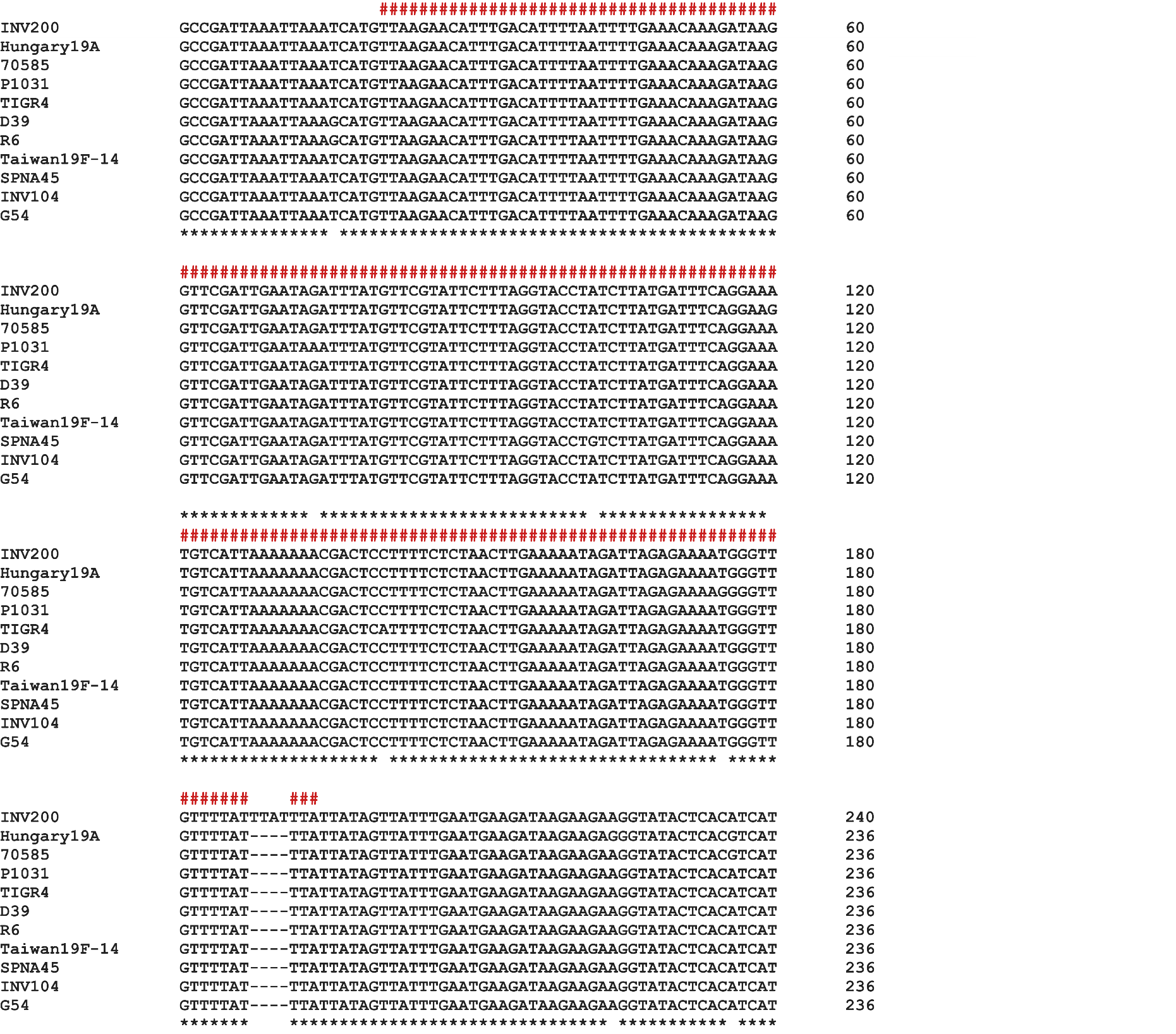


# Appendix Figure S1 - Conservation of F5 genomic region across pneumococcal species.

A multiple sequence alignment was performed with sequences from 11 pneumococcal strains: *Streptococcus pneumoniae* INV200, Hungary19A, 70585, P1031, TIGR4, D39, R6, Taiwan19F-14, SPNA45, INV104 and G54. The F5 region is highlighted with by a #.


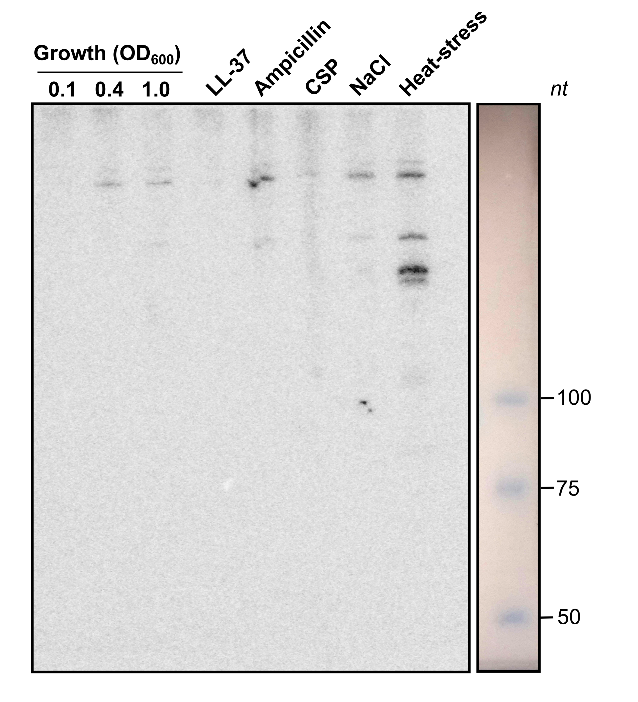


# Appendix Figure S2 – F5 Northern blot with RNA ladder.

F5 RNA levels were analyzed by northern blot analysis using RNA from standard growth conditions (OD600 0.1, 0.4 and 1.0 in C+Y media at 37°C), and different stress- and infection-relevant conditions (exponentially growing pneumococci were exposed to: 4 µg/mL antimicrobial peptide LL-37 for 30 minutes, 100 µg/mL ampicillin treatment for 30 minutes, competence induction with 0.4 µg/mL competence-stimulating peptide (CSP) for 12 minutes, sodium-chloride stress (0.5 M) for 30 minutes and heat-stress for 30 minutes at 42°C). Blot displays full membrane shown in Fig 1C, including an RNA ladder.


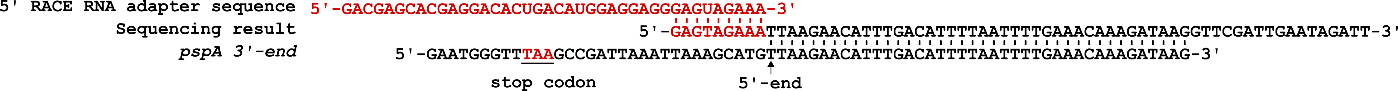


# Appendix Figure S3 - Mapping the 5’-end of the F5 RNA fragment by 5’ RACE analysis.

**S**anger sequencing of the cDNA PCR fragment from 5’ RACE analysis revealed the 5’-end of the F5 RNA fragment to be located 20 nucleotides downstream the *pspA* stop codon**.**


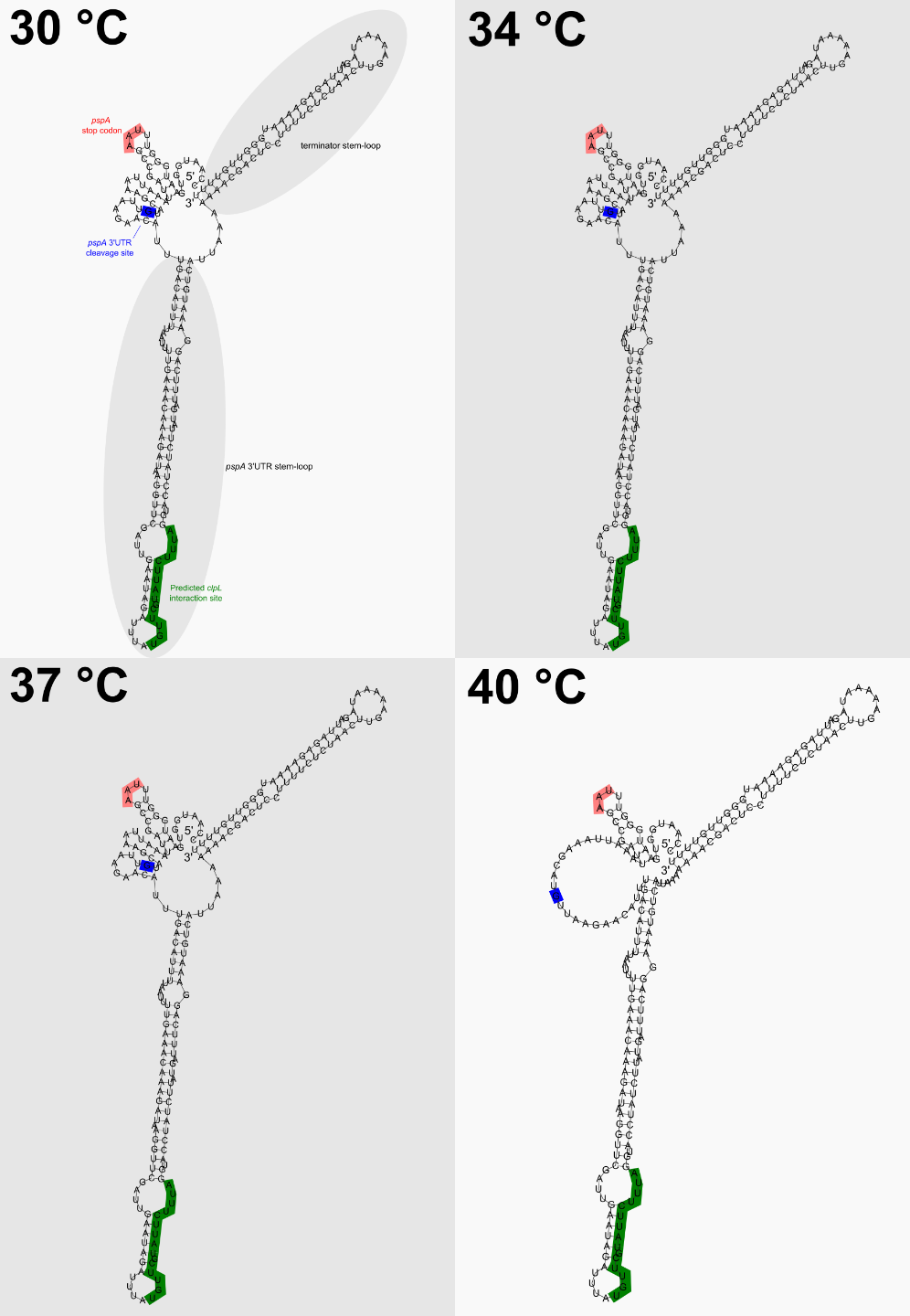


Appendix Figure S4 – Predicted secondary structure of the pspA 3’UTR region across temperatures. Secondary structure predictions were generated using the RNAfold webserver (http://rna.tbi.univie.ac.at/cgi-bin/RNAWebSuite/RNAfold.cgi) with default parameters at 30°C, 34°C, 37°C, and 40°C. Key features are highlighted: the *pspA* stop codon (red), the 3′ UTR cleavage site (blue), and the predicted *clpL* interaction site (green).

**
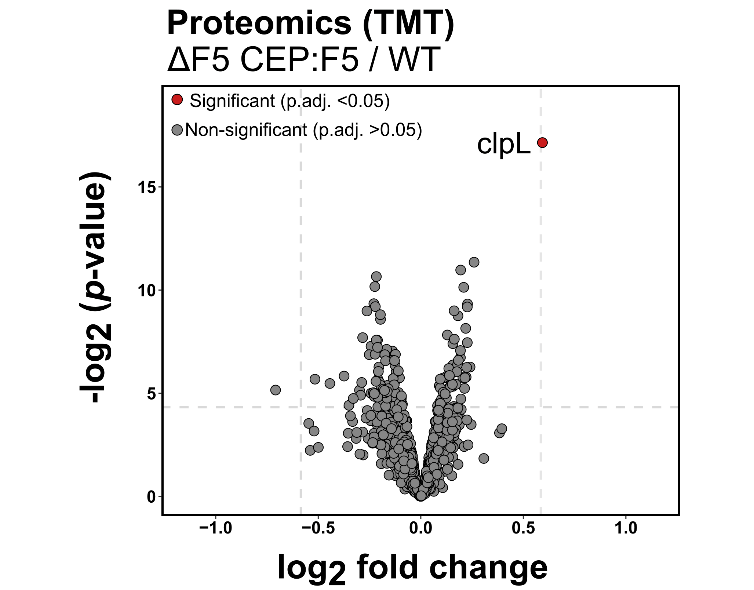
**

# Appendix Figure S5 - Quantitative proteomics of D39 Δ3UTR CEP:3UTR compared to wild type.

Quantitative mass spectrometry shows significant upregulation of the protein chaperone ClpL in the Δ*3UTR* CEP:*3UTR* strain compared to wild type. Samples were harvested from cultures grown in C+Y medium at 40°C to OD_600_ 0.5. Significantly (p.adj. <0.05) and non-significantly (p.adj. >0.05) regulated proteins are depicted with a red and grey dot, respectively.

**
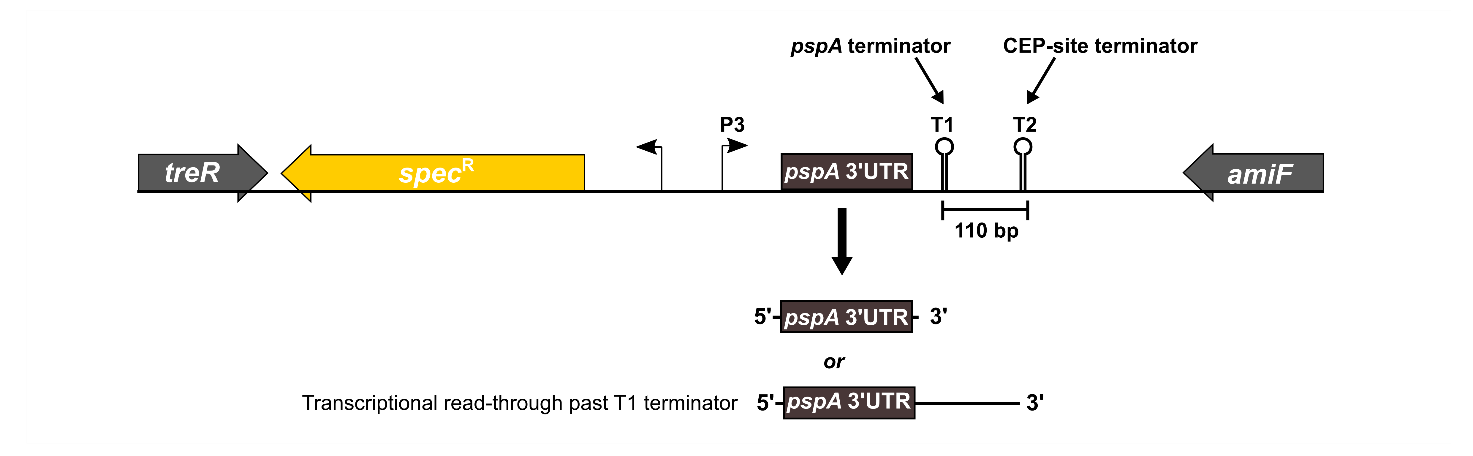
**

# Appendix Figure S6 - Read-through transcription at the CEP-site.

Illustration of the chromosomal expression platform (CEP) site with overexpression of the *pspA*-3’ UTR from the constitutive active promoter (P3), depicting a scenario for transcriptional read-through resulting in termination at the CEP-site terminator (T2).

**
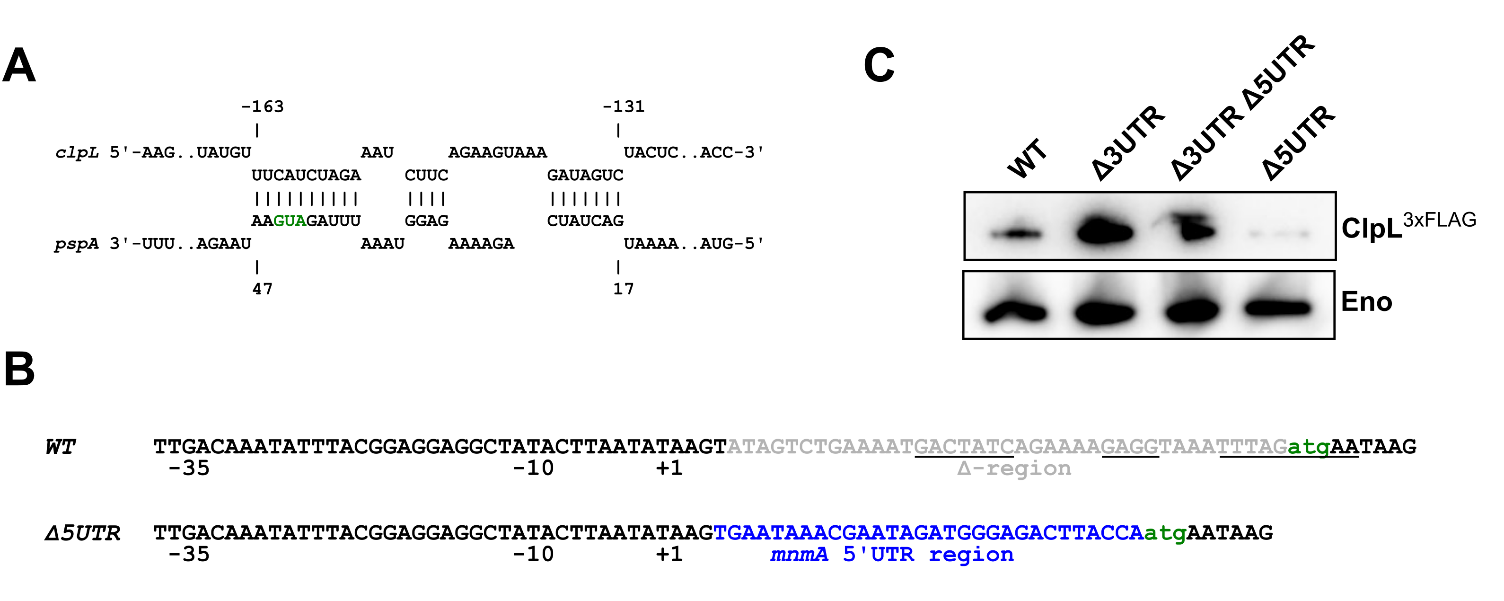
**

Appendix Figure S7 – Western blot analysis of ClpL expression in a *pspA*-5′ UTR deletion mutant (Δ5UTR).

A. IntaRNA predicts an interaction between the *pspA* minimal construct and the *clpL* 5′ UTR region.

B. To investigate the role of this region in regulating *clpL* expression, we constructed a deletion mutant in which most of the *pspA* 5′ UTR (grey) was replaced with a sequence encompassing the ribosome binding site (RBS) from the 5′ UTR of the neighboring gene *mnmA* (blue). Regions predicted to engage in base-pairing interactions with the *clpL* mRNA are underlined. The transcription start site is indicated as +1, while the predicted −10 and −35 promoter elements are also marked. Start codon is highlighted in green.

C. ClpL expression was assessed by western blot analysis at 30°C in the following strains: WT *clpL*-3×FLAG, Δ*5UTR* *clpL-3×flag*, Δ*3UTR* *clpL-3×flag*, and Δ*5UTR* Δ*3UTR* *clpL-3×flag*. Western blot was carried out using monoclonal mouse anti-FLAG M2 antibodies and rabbit anti-enolase antibodies as loading control.

**
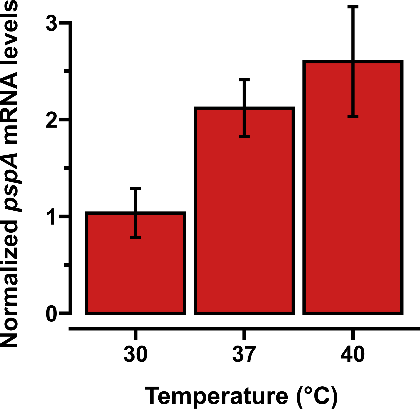
**

Appendix Figure S8 - Expression of *pspA* mRNA at different temperatures. RNA from exponentially growing D39 wild-type cultures (OD_600_ 0.3) at 30, 37 and 40°C was used for evaluating *pspA* mRNA expression by Reverse-Transcriptase quantitative PCR (RT-qPCR). RNA levels were normalized to *gyrA* mRNA expression. Data represent mean of three biological replicates. Error bars represent standard deviations.


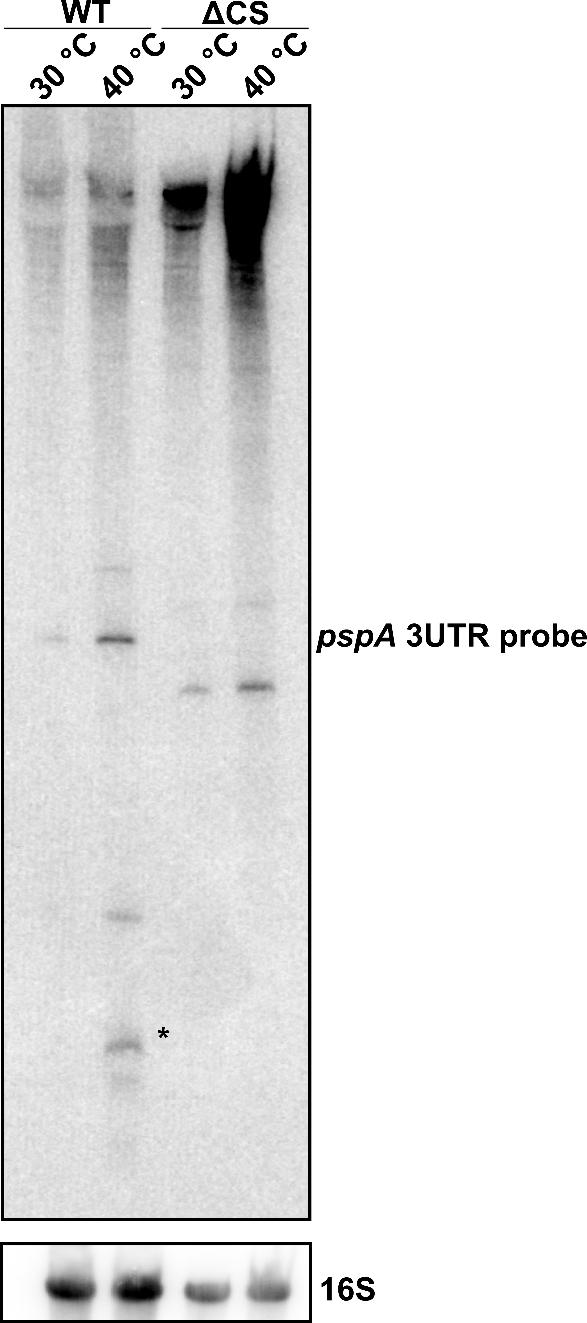


Appendix Figure S9 – Northern blot analysis of *pspA* mRNA and cleavage products in wild-type (WT) and ΔCS mutant strains at 30°C and 40°C. Total RNA was extracted from C+Y cultures grown at the indicated temperatures and probed for pspA products using a probe targeting the 3′ UTR. 16S rRNA was used as a loading control. The blot reveals temperature-dependent and cleavage-site-dependent formation of a pspA 3′ UTR-derived cleavage product (denoted by an asterisk).


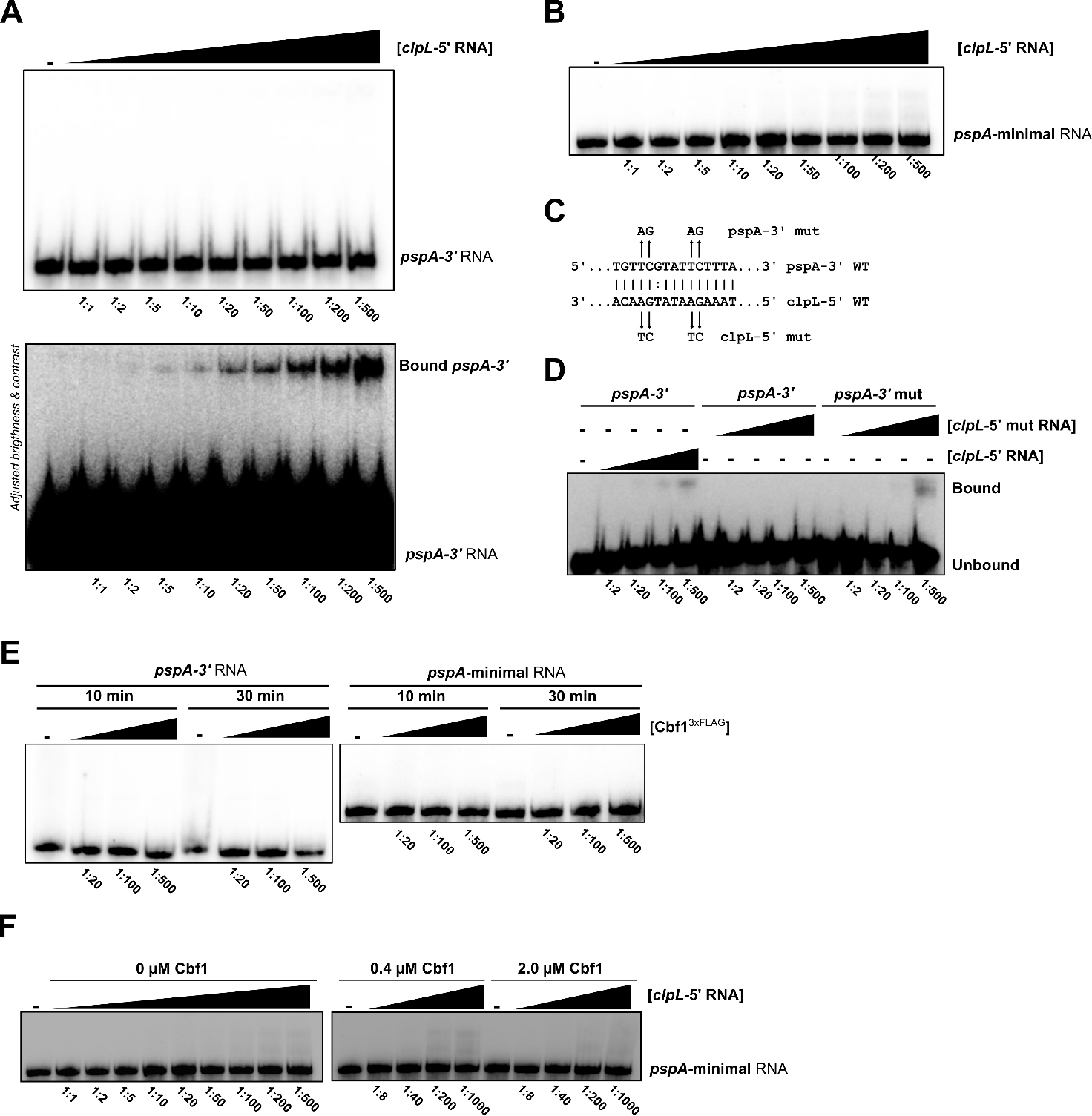


Appendix Figure S10 - Investigation of *in vitro* interaction between the *clpL*-5’ RNA and *pspA*-3’ *RNA* or *pspA* minimal constructs by gel shift assays.

A, B. Electrophoretic mobility shift assays (EMSAs) using either (A) in vitro *pspA*-3’ RNA (165 nt) and *clpL*-5’ RNA (287 nt) (B) or *pspA*-minimal RNA (368 nt) and *clpL*-5’ RNA (287 nt). Gel shift assay reveals a weak interaction between the *pspA*-3’ and *clpL*-5’ transcripts. The labeled RNAs were used at a final concentration of 2 nM.

C. Illustration of the compensatory mutations introduced into the in vitro *pspA*-3’ RNA and *clpL*-5’ RNA, within the predicted *clpL*-*pspA* interaction site (*pspA*- 3’ mut and *clpL*-5’ mut).

D. Gel shift assays were performed with the following combinations of in vitro transcripts: *pspA*-3’ RNA + *clpL*-5’ RNA, *pspA*-3’ RNA + *clpL*-5’ mut RNA, and *pspA*-3’ mut RNA + *clpL*-5’ mut RNA. Mutations to the predicted interaction site abolishes the very weak interaction, but compensatory mutations rescue it. The labeled RNAs were used at a final concentration of 2 nM.

E. Slightly faster migration was observed for the *pspA*-3’ RNA in a gel shift assay using purified Cbf1^3xFLAG^ and in vitro *pspA*-3’ or *pspA*-minimal RNA. However, no shift was observed, indicating no stable interaction between the transcripts and Cbf1^3xFLAG^. The labeled RNA was used at a final concentration of 2 nM.

F. No indication of a role for Cbf1 in mediating RNA-RNA interactions, as depicted in gel shift assays using *pspA*-minimal RNA and *clpL*-5’ RNA, with Cbf1 pre-treatment (0-2 µM) of the *pspA*-minimal RNA.


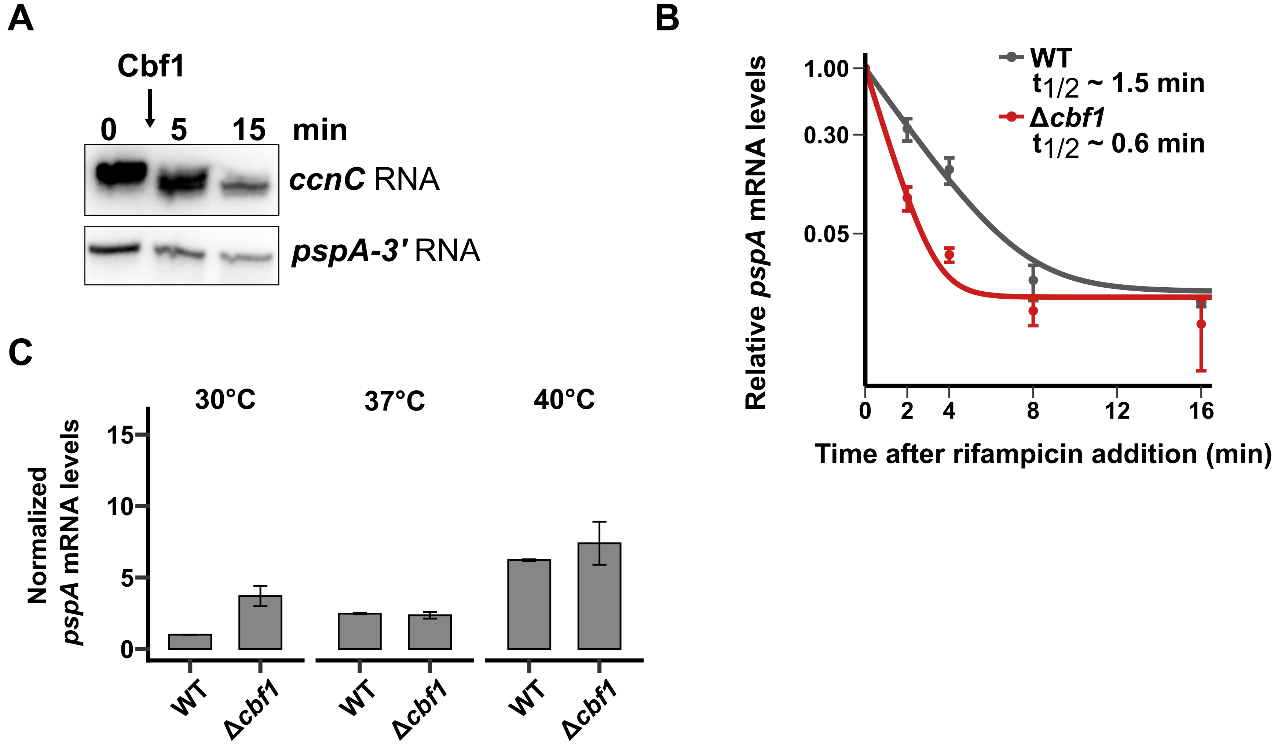


# **Appendix Figure S11 – Analysis of Cbf1-mediated of** *pspA*-3’-end trimming.

A. Cbf1 incubation assay with *in vitro* transcribed *pspA*-3’ RNA and *ccnC* RNA (99 nt) (control, previously identified to be 3’-end trimmed by Cbf1, as described in (1)). Both the *ccnC* RNA and the *pspA*-3’ RNA treated with Cbf1-3xFLAG showed slightly faster migration on a 8% polyacrylamide gel, indicating 3’-end trimming.

B. The stability of the *pspA* mRNA in the WT and Δ*cbf1* strains was evaluated by a rifampicin assay.

C. Steady-state *pspA* mRNA levels in WT and Δ*cbf1* strains at 30°C, 37°C, and 40°C were assessed by RT-qPCR, with gene expression normalized to *gyrA* mRNA.


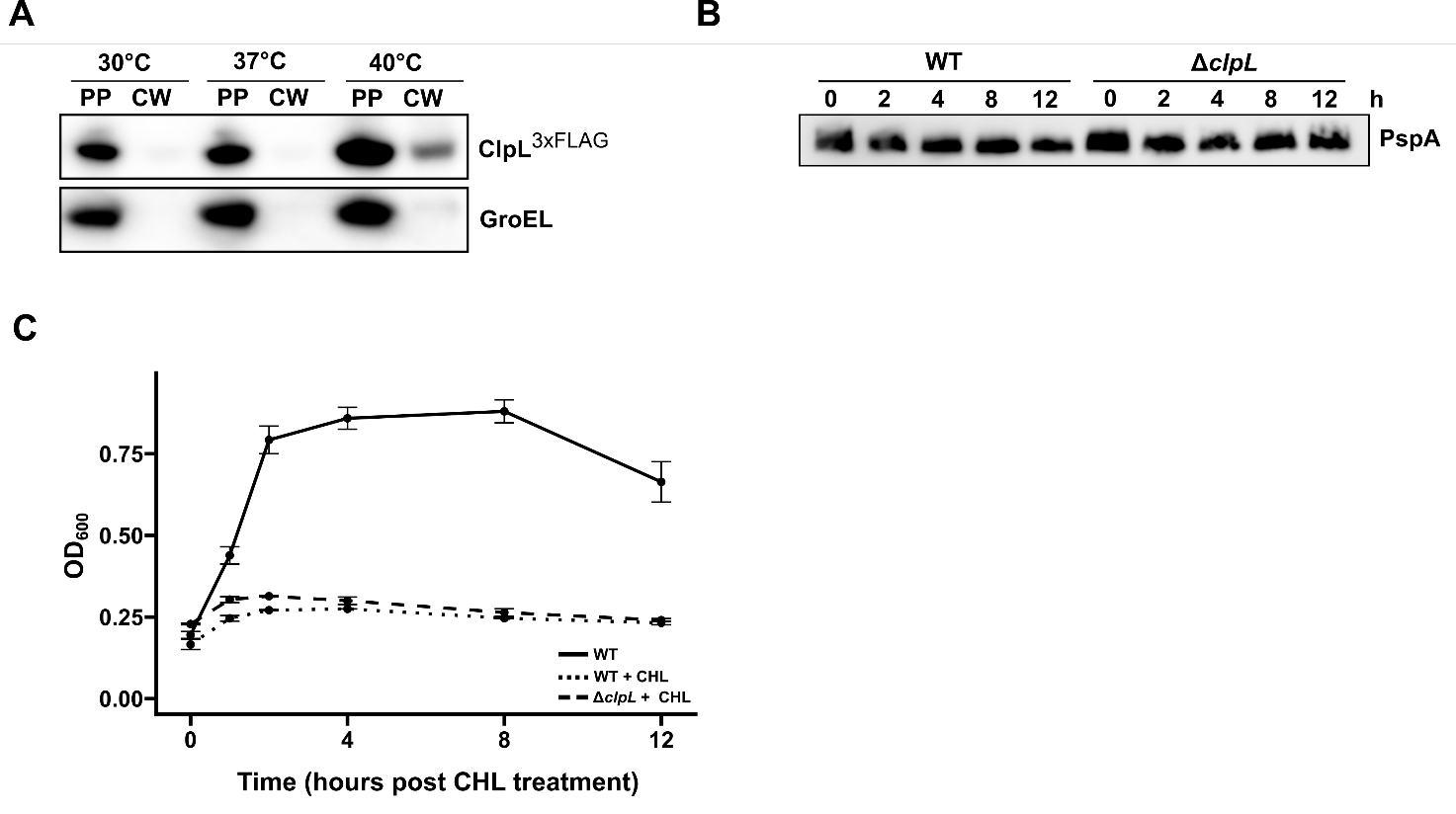


Appendix Figure S12 – Western blot analysis of PspA stability and ClpL localization.

A. *Streptococcus pneumoniae* D39 WT or Δ*3UTR* was grown in C+Y medium at 37°C to the early exponential growth phase, before being transferred to 30, 37 or 40°C for 60 minutes. ClpL levels in subcellular fractions of *S. pneumoniae* were analyzed by Western blot analysis (PP: protoplast, CW: cell wall).

B. *Streptococcus pneumoniae* D39 WT or Δ*clpL* was grown in C+Y medium at 40°C to the early exponential phase, followed by protein synthesis inhibition with 10 µg/mL chloramphenicol (CHL) for up to 12 hours. PspA levels were analyzed by Western blot, with SDS-PAGE gels loaded from equal OD unit lysates. in

C. Growth curve showing the inhibitory effect of chloramphenicol (CHL) on pneumococcal growth.

Appendix Table S1 – Strain list. List of all strains used in the study (separate supplementary file).

Appendix Table S2 – Oligo list. List of all oligonucleotides used in the study (separate supplementary file).

z

Appendix Table S3 – Generation times of D39 WT and Δ*3UTR* strains at 30, 37 and 40 °C.

Generation times were estimated by fitting linear models to the exponential phase of OD growth curves (4 replicates per condition) using the R package growth rates (version 0.8.5). Statistical analysis was performed using a two-way ANOVA, followed by Tukey-adjusted pairwise comparisons to assess differences between strains at each temperature.

| **Temperature (°C)** | **Strain** | **Generation time (min)** | **95% CI** | **p-value (Δ3UTR vs WT)** | **Significance** |
| --- | --- | --- | --- | --- | --- |
| 30 | WT | 59.9 | [58.3,61.5] | 0.029 | * |
| 30 | Δ3UTR | 62.4 | [60.8,64.0] |  |  |
| 37 | WT | 32.6 | [31.0,34.2] | 0.197 | ns |
| 37 | Δ3UTR | 34.1 | [32.5,35.7] |  |  |
| 40 | WT | 35.3 | [33.7,36.9] | 0.179 | ns |
| 40 | Δ3UTR | 33.8 | [32.1,35.4] |  |  |

# **Appendix Table S4 –** Top five predicted interactions between the *pspA* and *clpL* mRNAs, as predicted by IntaRNA.

Predictions were generated using the IntaRNA web server (https://rna.informatik.uni-freiburg.de/IntaRNA/Input.jsp) with default parameters. For reference, the *clpL* mRNA has a start codon at position 230 and a stop codon at position 2333. The *pspA* mRNA starts at position 43 and ends at position 1900. The table lists the five highest-scoring predicted interactions between the two transcripts.

| **#** | **Query** | **Start (clpL)** | **End (clpL)** | **Start (pspA)** | **End (pspA)** | **Energy (kcal/mol)** | **Predicted interaction** |
| --- | --- | --- | --- | --- | --- | --- | --- |
| 1 | *pspA* mRNA | 1592 | 1613 | 1017 | 1039 | -20.56 | 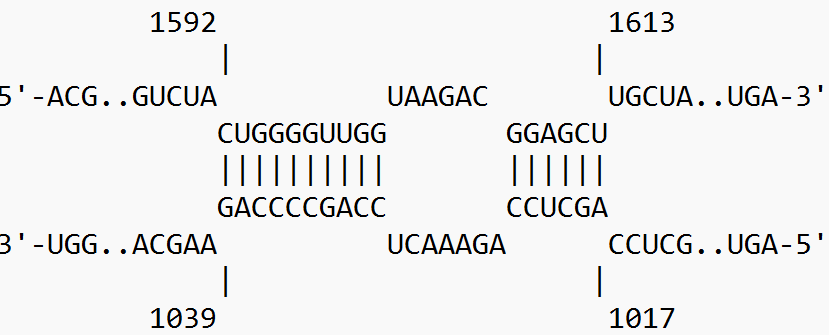 |
| 2 | *pspA* mRNA | 1361 | 1393 | 386 | 415 | -16.67 | 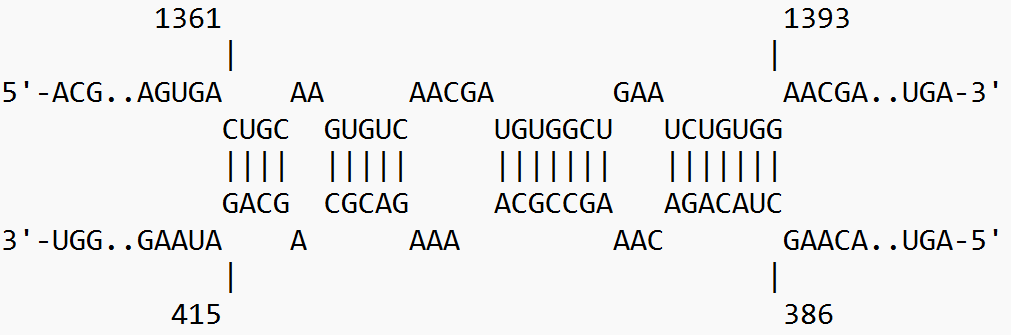 |
| 3 | *pspA* mRNA | 224 | 238 | 1982 | 1996 | -12.39 | 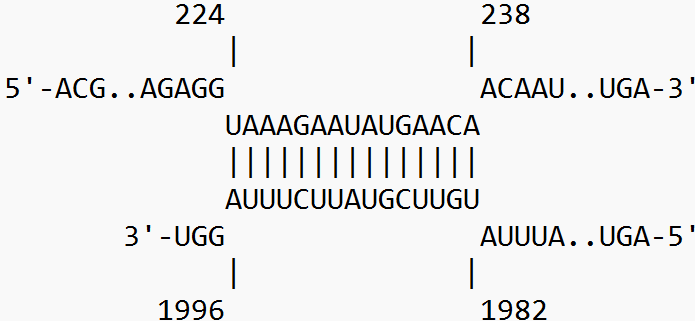 |
| 4 | *pspA* mRNA | 876 | 892 | 501 | 517 | -11.82 | 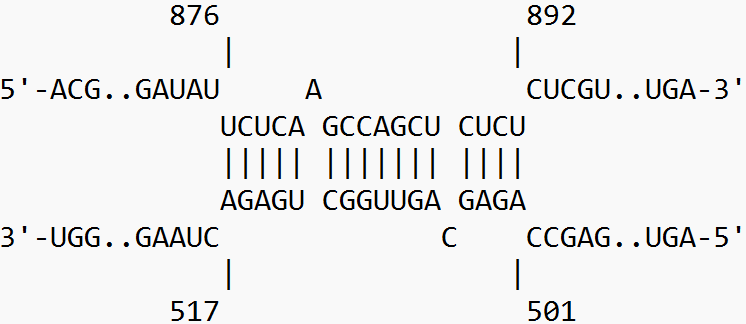 |
| 5 | *pspA* mRNA | 1048 | 1079 | 1722 | 1747 | -11.78 | 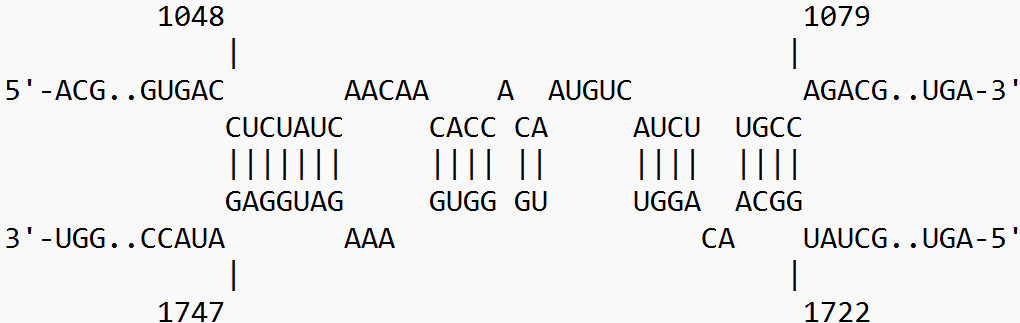 |

# Appendix references

(1) Hör J, Garriss G, Di Giorgio S, Hack L-M, Vanselow JT, Förstner KU, et al. Grad-seq Hör J, Garriss G, Di Giorgio S, Hack L-M, Vanselow JT, Förstner KU, Schlosser A, Henriques-Normark B, Vogel J (2020a) Grad-seq in a Gram-positive bacterium reveals exonucleolytic sRNA activation in competence control. *The EMBO journal* 39: e103852-e103852
